# Examining Relationships Between Groundwater Nitrate Concentrations in Drinking Water and Landscape Characteristics to Understand Health Risks

**DOI:** 10.1029/2021GH000524

**Published:** 2022-05-01

**Authors:** Q. F. Hamlin, S. L. Martin, A. D. Kendall, D. W. Hyndman

**Affiliations:** ^1^ Department of Earth and Environmental Sciences Michigan State University East Lansing MI USA; ^2^ Department of Geosciences School of Natural Sciences and Mathematics University of Texas at Dallas Richardson TX USA

**Keywords:** nitrate, groundwater, water quality, cancer, nitrogen, drinking water

## Abstract

Nitrate ingested from drinking water has been linked to adverse health outcomes (e.g., cancer, birth defects) at levels as low as ∼2 mg/L NO_3_‐N, far below the regulatory limits of 10 mg/L. In many areas, groundwater is a common drinking water source and may contain elevated nitrate, but limited data on the patterns and concentrations are available. Using an extensive regulatory data set of over 100,000 nitrate drinking water well samples, we developed new maps of groundwater nitrate concentrations from 76,724 wells in Michigan's Lower Peninsula, USA for the 2006–2015 period. Kriging, a geostatistical method, was used to interpolate concentrations and quantify probability of exceeding relevant thresholds (>0.4 [common detection limit], >2 mg/L NO_3_‐N). We summarized this probability in small watersheds (∼80 km^2^) to identify correlated variables using the machine learning method classification and regression trees (CARTs). We found 79% of wells had concentrations below the detection limit in this analysis (<0.4 mg/L NO_3_‐N). In the shallow aquifer (focus of study), 13% of wells exceeded 2 mg/L NO_3_‐N and 2% exceeded the EPA maximum contaminant level of 10 mg/L. CART explained 40%–45% of variation in each model and identified three categories of critical correlated variables: source (high agricultural nitrogen inputs), vulnerable soil conditions (low soil organic carbon and high hydraulic conductivity), and transport mechanisms (high aquifer recharge). These findings add to the body of literature seeking to identify communities at risk of elevated nitrate and study associated adverse health outcomes.

## Key Points

- Groundwater nitrate concentrations were mapped using over 100,000 samples in Michigan's Lower Peninsula for 2006–2015
- Concentrations were linked with a suite of potential driver variables using classification and regression trees
- Elevated nitrate was related to source (agricultural nitrogen inputs), vulnerability (soil conditions), and transport (aquifer recharge)

## 1 Introduction

Nitrate in drinking water has been identified as a pollutant that can be hazardous to human health since the mid‐1940s, when nitrate ingestion from well water was linked to fatal blue baby syndrome (Comly, 1945; Faucett & Miller, 1946; Ferrant, 1946). Since then, regulatory agencies in the US and World Health Organization (WHO) have imposed similar limits of 10 mg/L NO_3_‐N (US) and 50 mg/L NO_3_ (WHO, or 11.3 mg/L NO_3_‐N) for drinking water (USEPA, 2021; WHO, 2017). Epidemiological reviews (Ward et al., 2005, 2018) identified multiple studies linking drinking water with nitrate below these regulatory limits to a variety of cancers (Inoue‐Choi et al., 2015; Jones et al., 2016; McElroy et al., 2008; Schullehner et al., 2018; Stayner et al., 2021; Ward et al., 2010) and adverse pregnancy outcomes (Brender et al., 2013; Coffman et al., 2021; Holtby et al., 2014; Manasaram et al., 2006; Sherris et al., 2021; Weyer et al., 2014), although some studies report no association (Mattix & Winchester, 2007; Mueller et al., 2004; Waller et al., 2010; Winchester et al., 2009). Increased risk (expressed via odds ratio or hazard ratio) could be found at concentrations as low as 0.7–5 mg/L NO_3_‐N (Espejo‐Herrera, Gracia‐Lavedan, Boldo, et al., 2016; Espejo‐Herrera, Gracia‐Lavedan, Pollan, et al., 2016; Fan & Steinberg, 1996; Holtby et al., 2014; Inoue‐Choi et al., 2015; Schullehner et al., 2018; Temkin et al., 2019). As these health risks emerge, it is increasingly important to understand what environmental conditions lead to high drinking water nitrate, particularly at levels below current regulatory standards.

Groundwater is a critical resource in the United States, providing drinking water to over one third of the US population in 2010 (DeSimone et al., 2015). Available data shows many agricultural areas of the United States have high groundwater nitrate concentrations, with 3.6% of public water systems using groundwater with EPA violations from 1994 to 2015 (Pennino et al., 2017, Table S4) and 1% of the nation's 43 million private well users are in regions where violations are expected (DeSimone et al., 2015; Ransom et al., 2021). Private drinking water wells are used by 14.5% of United States households (Johnson et al., 2019). Michigan, a midwestern state, has the most domestic wells of any state in the United States with almost 2.5 million (Johnson et al., 2019). However, broad accessibility to data for users to analyze risks below regulatory levels is limited due to variability in regulations combined with the effort, cost, and privacy concerns related to sampling private drinking water wells. In Michigan, as part of a mandated groundwater monitoring program (Michigan Compiled Law 324.8713 [MI MCL 324.8713]), chemical analyses are mandated when new residential wells are drilled (Michigan Rule 562.412 [MI R 562.412]). Moreover, permits for new development will be rejected if concentrations exceed half the maximum contaminant level (MCL) of 10 mg/L NO_3_‐N and are expected to rise due to factors such as geology and land use (MI R 562.414). These requirements resulted in an extensive government‐maintained database of groundwater chemistry data for Michigan.

Nitrate concentrations in the subsurface are highly heterogeneous, related to variable loading pulses, geologic properties, and travel times through aquifers (Böhlke, 2002; Rivett et al., 2008; Schilling & Jacobson, 2010; Van Meter & Basu, 2015). Nitrate in groundwater is primarily sourced from anthropogenic alterations/additions to the global nitrogen cycle, including fertilizer, manure, septic systems, fixation from legumes, and atmospheric deposition (Galloway et al., 2004; Vitousek et al., 1997). Variable groundwater travel times create a time lag or legacy, complicating the risk of contaminated drinking water as nitrate from surface inputs may take decades to propagate through the system (Fenton et al., 2017; Kim et al., 2020; Martin et al., 2017; Van Meter & Basu, 2015; Vero et al., 2018). It is critical to identify populations at risk and adapt current management practices to mitigate future threats to health (Ascott et al., 2021; Hansen et al., 2017; Martin et al., 2021).

The complexity of the groundwater system and challenge of wide‐spread, routine sampling has led researchers to use a variety of index‐based (e.g., DRASTIC and DRASTIC‐P, Aller, 1987; DRASTIC‐N, Voutchkova et al., 2021), statistical (e.g., logistic regression, Helsel & Hirsch, 1992; non‐linear regression, Nolan & Hitt, 2006), and process‐based modeling (e.g., ANSWERS, Beasley et al., 1980; HYDRUS‐1D, SCANVA, Hansen et al., 2016; Šimůnek & van Genuchten, 2008) methods to identify vulnerable aquifers (Liggett & Talwar, 2009; Machiwal et al., 2018). Recently, machine learning methods like classification and regression trees (CARTs) (Burow et al., 2010; Uddameri et al., 2020), boosted regression trees (Motevalli et al., 2019; Nolan et al., 2015; Ransom et al., 2017, 2021; Uddameri et al., 2020), and random forest (Messier et al., 2019; Nolan et al., 2014; Pennino et al., 2020; Tesoriero et al., 2017; Uddameri et al., 2020; Wheeler et al., 2015) have been used to link large datasets of nitrate samples with potential driver variables to predict concentrations at non‐sampled locations. These tree‐based algorithms assess the importance of different variables for nitrate concentrations, which can help decipher the underlying conditions that lead to elevated concentrations in a region. Studies have reported the most significant correlative variable categories are related to nitrogen inputs (Nolan et al., 2015; Nolan & Hitt, 2006; Pennino et al., 2020; Tesoriero et al., 2017), geologic properties (Messier et al., 2019; Motevalli et al., 2019), geochemical conditions (Ransom et al., 2017), and well depths (Uddameri et al., 2020; Wheeler et al., 2015), although all studies utilize a combination of key variables. These tree‐based methods can also be used to both predict concentration in unsampled areas and identify populations at risk.

**Figure 1:**
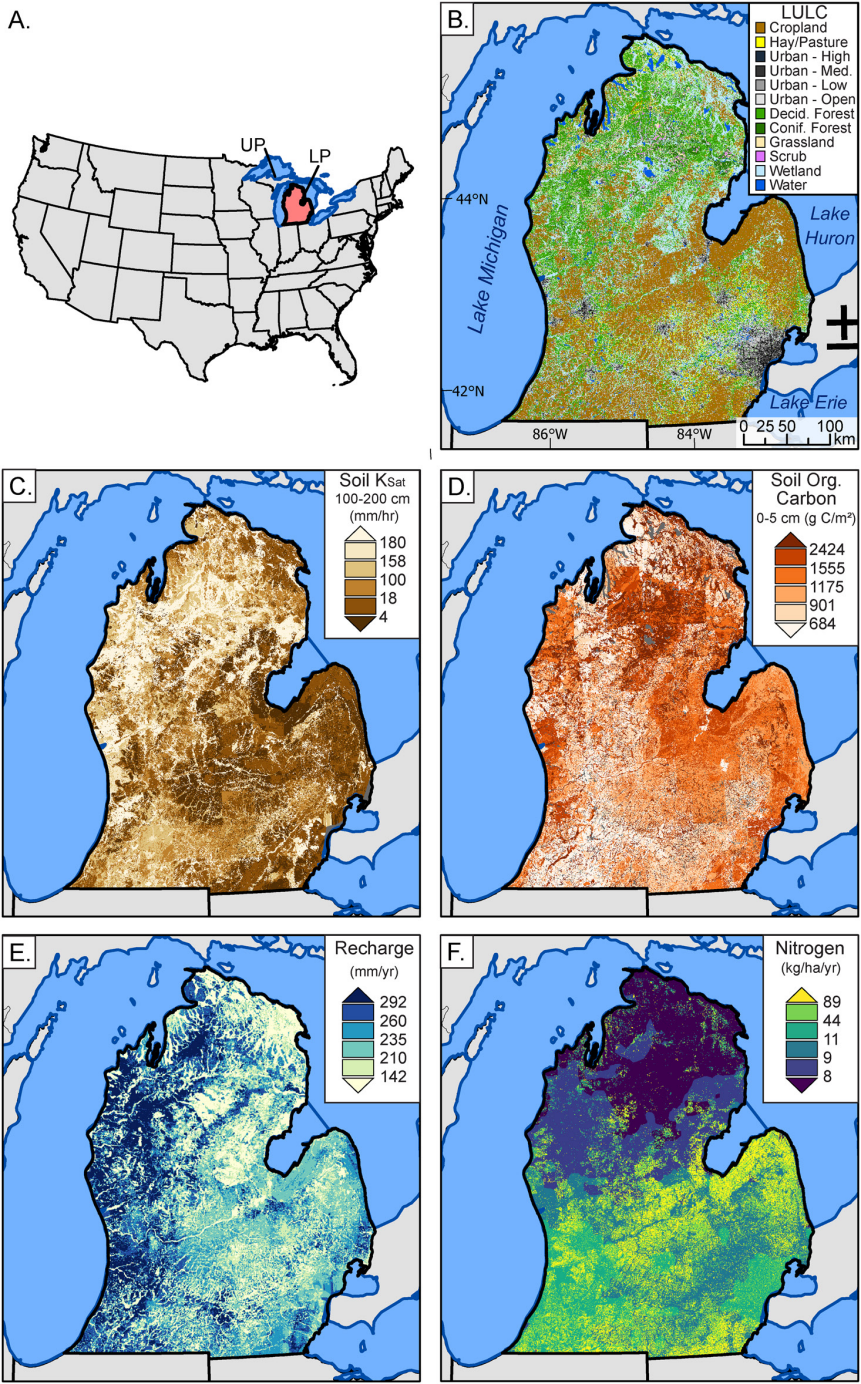
Study area with: (a) locator map within the Continental United States with Michigan's Lower Peninsula (study area) highlighted in red and labeled “LP”. The Upper Peninsula is labeled above “UP” and mostly obscured by the study area boundary, (b) land use land cover (National Land Cover Database, Homer et al., 2015), (c) soil hydraulic conductivity estimates (*K*_sat_), (d) soil organic carbon, (e) recharge, and (f) nitrogen inputs from SENSEmap (Hamlin et al., 2020a, 2020b). Breaks in legends are chosen using quantile intervals, where each color represents 16.7% of the data.

Here, we analyze potential drivers of groundwater nitrate concentrations with CART analysis using an extensive data set of nitrate measurements from drinking water wells across Michigan's Lower Peninsula (Figure 1). Over 100,000 samples from 76,724 unique wells were used to characterize and interpolate nitrate concentrations across the region using the geostatistical method Empirical Bayesian Kriging (EBK) (Hussain et al., 2010). We create maps of conservative exceedance probabilities for two concentration levels using EBK. By using kriging, we create a smooth surface of nitrate concentrations that reduces effects of noise and spatial clustering in the original data set. We then summarize this map at the small surface‐watershed (HUC12, U. S. Geological Survey [USGS], 2013) scale and analyze relationships among 89 variables describing soil and geologic properties, nitrogen loads, land use, and well characteristics using CART. CART, a tree‐based machine learning algorithm, illuminates correlated variables in an easily interpretable manner. Kriging reduces noise in the highly variable data followed by CART to analyze patterns. Our methods make use of the high data density and categorize nitrate concentration at a management‐appropriate scale (e.g., watershed rather than individual well). To our knowledge, this is the first public visualization and analysis of nitrate from Michigan's massive regulatory data set. The objectives of this work are to:

1. Map nitrate concentrations in Michigan's Lower Peninsula for the 2006–2015 period using over 76,000 unique drinking water wells with more than 100,000 samples
2. Assess potential drivers of nitrate concentrations using correlations between these values and landscape characteristics at the small watershed scale

With this extensive data set, we developed maps of groundwater nitrate concentrations and linked them to the physical characteristics and land management. An improved understanding of groundwater nitrate concentrations at lower thresholds and their potential drivers can help inform management strategies, quantify nitrate exposure, and identify areas of current and potentially future health concerns.

## 2 Methods

### 2.1 Study Area

Michigan is located in the Midwestern United States and bordered by four of the five Laurentia Great Lakes (Figure 1a). Due to data availability in the Upper Peninsula of Michigan, we limited our analyses to the state's 104,019 km^2^ Lower Peninsula (hereafter LP). Coarse‐textured glacial deposits cover much of the LP, with higher clay and silt percentages in the central and eastern LP that was deposited after prehistoric proglacial lakes drained. These Quaternary‐age glacial drift aquifers are commonly used for private drinking water wells in the region (Michigan Water Wells for Wellogic, 2019). Annual precipitation for the LP ranges from ∼700 to 1,100 mm/yr (PRISM Climate Group, 2012).

Land use within the LP ranges from urban areas and intensive agriculture to large swaths of forests and wetlands (Figure 1b, Homer et al., 2015). Metropolitan areas such as Detroit, Kalamazoo, Lansing, and Grand Rapids are home to most of Michigan's over 9 million residents (U.S. Census Bureau, 2010). Much of the land area of the southern LP is managed for agriculture in major row‐ and field‐crops, including corn‐soy rotations, wheat, and hay, with orchards, vineyards, and other fruit crops along the Lake Michigan coastal region (USDA (2012), USDA Census of Agriculture). North of 44°N, the LP is largely forested and more sparsely inhabited.

### 2.2 Geologic and Landscape Characteristics

We identified several geologic and landscape characteristics that potentially drive or modulate groundwater nitrate concentrations. Below, we describe the datasets and methods used to create this geographical information for further use in our analyses. Summaries of data sources can be found in Table 1.

**Table 1:** Data Sources

Data type	Data source	Author	Time	Spatial resolution
Groundwater nitrate concentration	Well Chemistry	MI EGLE	2006–2015	Well Points
Well properties	Wellogic	MI EGLE	2006–2015	Well Points
Nitrogen surface inputs	SENSEmap	Hamlin et al. (2020a, 2020b)	2008–2015	30 m
Nitrogen groundwater inputs	SENSEflux	Luscz et al. (2017), Martin et al. (2021)	2008–2015	120 m
Soil properties	gSSURGO	USDA Natural Resources Conservation Service, Schaap et al. (2001)		10–30 m
Aquifer hydraulic conductivity	Wellogic (Derived)	Wellogic, Farrand and Bell (1982)		120 m
Recharge	Modeled	Hyndman et al. (2007)	2010	120 m
Land use/land cover	National Land Cover Database	Homer et al. (2015)	2011	30 m
Water table depth	Interpolated from Wellogic	Martin et al. (2021), Supplemental	1980–2016	30 m
Glacial drift thickness	USGS Map 3392	Soller and Garrity (2018)		500 m
Crop variables	Cropland data layer	USDA National Agricultural Statistics Service	2010–2015	30 m

*Note.*A summary of data types, sources, authors, representative time, and spatial resolution. Detailed variables found in each group can be found in Table S2. MI EGLE is the abbreviation for Michigan Department of Environment, Great Lakes, and Energy.

*Soil:*We used the USDA gSSURGO database (Soil Survey Staff, 2020) to create maps of 28 soil characteristics. *Plant*‐*available water storage* (m^3^ water/m^3^ soil) and *soil organic carbon* (SOC) (g C/m^3^ soil) were derived from the value‐added attributes tables (valu1 table) distributed with gSSURGO. *SOC* content is the mass of organic carbon per unit volume of soil, for the six available layers (0–5, 5–20, 20–50, 50–100, 100–150, and 150+ cm). *Soil textural* characteristics (% sand, % silt, % clay) define the particle size distribution within the soil column. Saturated vertical *hydraulic conductivity* (*K*_Sat_, mm/hr), which relates to how quickly water can move through the soil profile, was estimated from soil textural characteristics using the v1 ROSETTA database (Schaap et al., 2001). Soil textures and *K*_Sat_ were computed from the gSSURGO data for four layers (0–20, 20–50, 50–100, and 100–200 cm).

*Aquifer:*Aquifer *hydraulic conductivity* (HK) was estimated by the Michigan State University Remote Sensing and GIS group using well log descriptions of sediment texture and pump tests, as reported in the Michigan Wellogic Database (Michigan Waterwells for WELLOGIC dataset, 2019). To extend these point‐based measurements to the rest of Michigan, we used the geometric mean of well‐based K for each Quaternary geology polygon (Farrand & Bell, 1982). We used the arithmetic average of HK values for other polygons of similar geologic class for polygons without wells present.

Groundwater *recharge* was estimated for the study area following an approach described in Luscz et al. (2017), similar to methods reported by the USGS (Holtschlag, 1997). Briefly, linear relationships between observed precipitation and predicted recharge were calculated using Landscape Hydrology Model (LHM) outputs for a watershed in Michigan (Hyndman et al., 2007; Kendall, 2009; Wiley et al., 2010). Modeling was performed over a 28‐year period for approximately 20,000 km^2^ within and around the Muskegon HUC8 watershed in west‐central LP. This watershed has diverse geologic and land use characteristics that represent conditions across the remainder of the LP. Within all five major land use classes (urban, agricultural, grass, deciduous, coniferous), linear regressions were developed to quantify the fraction of annual precipitation that becomes aquifer recharge as a function of saturated soil hydraulic conductivity.

Background groundwater nitrate concentrations are extremely low, ranging from 0 to 0.27 mg/L NO_3_‐N with a median of 0 mg/L NO3‐N (Dumouchelle et al., 1987). Groundwater resources in Michigan include areas with both oxic and anoxic conditions in shallow and deep groundwater, though deep groundwater tends to be more frequently anoxic than shallow (Erickson et al., 2021). This study does not explicitly incorporate redox conditions, due to data limitations.

*LULC and Agriculture:*Land Use/Land Cover (LULC) variables were summarized from the 2011 National Land Cover Database (NLCD), shown in Figure 1a (Homer et al., 2015), and USDA Cropland Data Layers (CDLs). Both individual land use classes as reported in NLCD and aggregated land use classes (e.g., urban, forest, agriculture, wetland) were used within our analyses. LULC classes were represented as percent of total watershed area. Cropland variables derived from the CDL included the median percent of land in corn, soy, or other agriculture from 2010 to 2015, the available years within our study period (USDA‐NASS, 2015).

*Nitrogen Inputs and Loading:*The Spatially Explicit Nutrient Source Estimate map (SENSEmap) provided estimates of total nitrogen inputs to the land surface, which quantifies point sources and six non‐point sources of nitrogen inputs to the landscape by synthesizing literature, remote sensing products, government records (e.g., US Census, US Agricultural Census), and modeling products (e.g., estimates of county level fertilizer loads; Hamlin et al., 2020a, 2020b; Luscz et al., 2015). Non‐point sources include atmospheric deposition, chemical agricultural fertilizer, manure, chemical non‐agricultural fertilizer, septic tanks, and nitrogen fixation from legumes. SENSEflux (Martin et al., 2021) is an expanded and updated model based on previous work by Luscz et al. (2017) that implements a statistical transport model to quantify the amount of nitrogen that survives transport to the Great Lakes coastline after attenuation along surface and groundwater pathways. Here, we use SENSEmap inputs to the land surface (kg/ha/yr) and SENSEflux estimates of mobile nitrogen in groundwater (kg/ha/yr), for all sources. Our estimates are representative of the 2008–2015 period, consistent with this analysis' 2006–2015 period.

### 2.3 Drinking Water Well Data

The Michigan Department of Environment, Great Lakes, and Energy (EGLE) provides a publicly available database of drinking water well information called Wellogic. Information in this database has been digitized from well boring logs and contains all wells drilled since 1996. Wells drilled prior to 1996 have partial records due to variability in county‐by‐county archival digitization efforts. Data recorded includes drilling date, well screen intervals, and aquifer properties. In this study, we utilized both the spatial location of the wells and aquifer information. Drinking water well chemistry data was obtained via a Freedom of Information Act request from EGLE in December 2019. This database included over 3.6 million samples of various chemicals from municipal and private drinking water wells across the state collected from 1984 to 2019. Addresses included in the database were inconsistently documented and lacked additional spatial reference such as latitude‐longitude coordinates. Quality control/quality assurance (QA/QC) procedures were developed to standardize address formats using OpenRefine and were then assigned coordinates using geocoding in ArcGIS Pro 2.5 (ESRI, 2020a). After QA/QC procedures, 175,090 unique wells with 511,065 samples remained in the 1984–2019 data set. For this study, only samples collected from 2006 to 2015 were used to limit temporal “noise” and focus on a time period represented by corresponding data (e.g., SENSEmap and SENSEflux). This resulted in 121,492 samples from 76,724 unique wells for our final data set. Wells with multiple samples available in the study period (23%) were represented by the geometric mean.

### 2.4 Interpolating Groundwater Nitrate Concentration With Kriging

We used kriging, a geostatistical method, to interpolate a continuous surface of groundwater nitrate concentrations, and surfaces of the probability of exceeding 0.4 mg/L (common detection limit in data set) and 2 mg/L NO_3_‐N (associated with increased health risk). By first using kriging in this analysis, we smooth the noise found in the large data set and reduce the effects of imbalanced sampling across the study area. Due to the large spatial extent, variable sampling density, and variation in concentration measurements, we used EBK in ArcGIS Pro 2.5 to automate the interpolation process (ESRI, 2020b). This method iteratively produces semivariograms for subsets of the data to tailor the kriging function to each neighborhood of points, thus removing user's manual control of fitting a semivariogram function to the entire data set (ESRI, 2020b; Hussain et al., 2010). Kriging was performed with 250 m cells using multiple simulated exponential semivariograms. Further details on kriging parameters and examples of semivariogram models can be found in Text S1, Figure S1, and Table S1 in Supporting Information S1.

Probability kriging computes the probability of a cell's kriging prediction to exceed a given threshold, and thus provides additional information beyond kriging predictions. The probability kriging option within EBK was used here to test two thresholds of exceedance: 0.4 (the most common detection limit in the data set, or lowest detectable concentration of nitrate by the analytical machine) and 2 (a value from the range of lower concentration risks; Ward et al., 2018; Temkin et al., 2019). Our samples represent either: (a) measurements taken from new or existing GW wells, or (b) public drinking water supplies. In either case, these wells have been developed to be safe drinking water sources and are thus likely to avoid both locations and depths within the aquifer with known high NO_3_ concentrations. In contrast, measurements below 5 mg/L NO_3_‐N do not trigger any state actions and are thus likely more representative of both current and future water supplies.

Although kriging produces estimates for every cell within the study area, estimation error rises with distance from data points. Thus, we only included kriging results within 3 km of a sample point (the correlation length as determined separately from simple kriging) to mask areas where kriging does not have sufficient data and estimates approach the data set mean rather than being influenced by nearby points.

### 2.5 Classification and Regression Trees (CART)

CART analysis was used to explore nonlinear statistical relationships between groundwater nitrate concentrations and potential driver variables (De’Ath & Fabricius, 2000). Although CART can be used for prediction, it is used here as a method for data exploration. We used *rpart* 4.1.15 in R version 3.6.0 to implement all CART analysis (R Core Team, 2019; Therneau et al., 2019). CART performs nested hierarchical “splits” to a data set using a single response variable and a suite of driver variables as inputs. CART starts with the entire data set and breaks the response variable into two groups based on thresholds in the driver variable that minimizes within‐group variance. Each resultant group is then split again (the split is referred to as a node) based on whichever driver next minimizes variance. These recursive splits create the inverted “tree” shape common to decision trees. CART stops splitting when the “pruning” criteria is reached. We specified that pruning would end when an individual split did not increase the performance by >3% (complexity parameter, cp > 0.03); above 3%, the final tree had very few splits, with little explanatory power, while below 3% the smallest nodes had very few members. Here, as our intent was to use CART to illuminate spatial patterns across watersheds and relationships to potential driver variables, we did not employ a test/validate/train architecture, but rather chose to maximize the statistical power within the data set and used our entire sample to train the tree. The final CART results include the optimal decision tree and terminal groups, a measure of variance explained (Proportional reduction in error [PRE]), and a list of “competitor” and “surrogate” options for each split. PRE, here reported relative to the training data set fit, behaves similarly to the coefficient of determination (*R* ^2^) used in linear regression, ranges from 0 to 1 and corresponds to the proportion of data set variance explained. Competitor splits are defined for each node and would divide the data into groups with a similar PRE, but can create different groups than the optimal split. Surrogate splits involve other drivers that would split the group in a similar manner to the optimal split. Analysis of competitor and surrogate splits can provide extended understanding of the correlation structure within the data, though it was not included here for brevity. Although CART can be optimized by fine‐tuning model parameters,we used a manual calibration for data exploration. If prediction is the intended use of CART outputs, additional model tuning would be required.

We summarized driver and response data at the watershed level using the USGS Watershed Boundary Data set Hydrologic Unit Code system; specifically, at the HUC12 scale, which for our study area includes 1,048 watersheds with an average area of ∼80 km^2^ (USGS, 2013). Within watersheds, summaries were compiled using medians for all variables except land use and soil texture, which were reported as areal proportions that sum to one. Nutrient source variables were also summarized with means. Median probability of threshold exceedance in each watershed was used as the response variable. Driver variables included representatives from all variables in Table 1. Detailed descriptions of all 89 included variables are shown in Table S1. Watersheds were only included in CART analysis if greater than 66% of the area had kriging estimates. This ensured that sufficient data was available to characterize groundwater nitrate conditions in the watershed, which resulted in 79% of the LP used in this analysis.

## 3 Results

### 3.1 Mapping Groundwater Nitrate Concentrations

**Figure 2:**
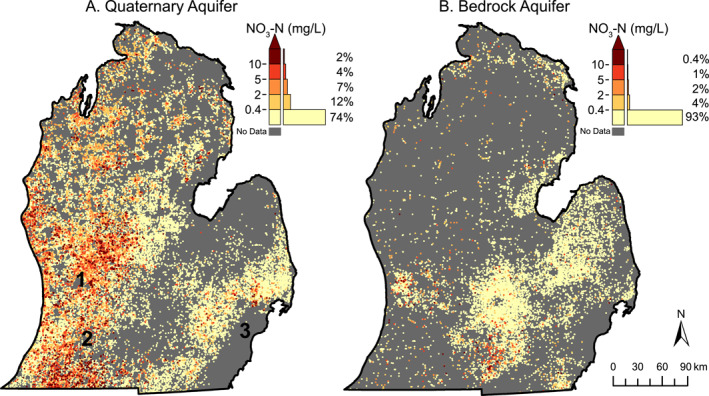
Concentrations of NO_3_‐N for drinking water wells sampled from 2006 to 2015. Legends show the distribution within each bin normalized to 100%. Black numbers denote general locations of major cities: 1) Grand Rapids, 2) Kalamazoo, and 3) Detroit.

**Figure 3:**
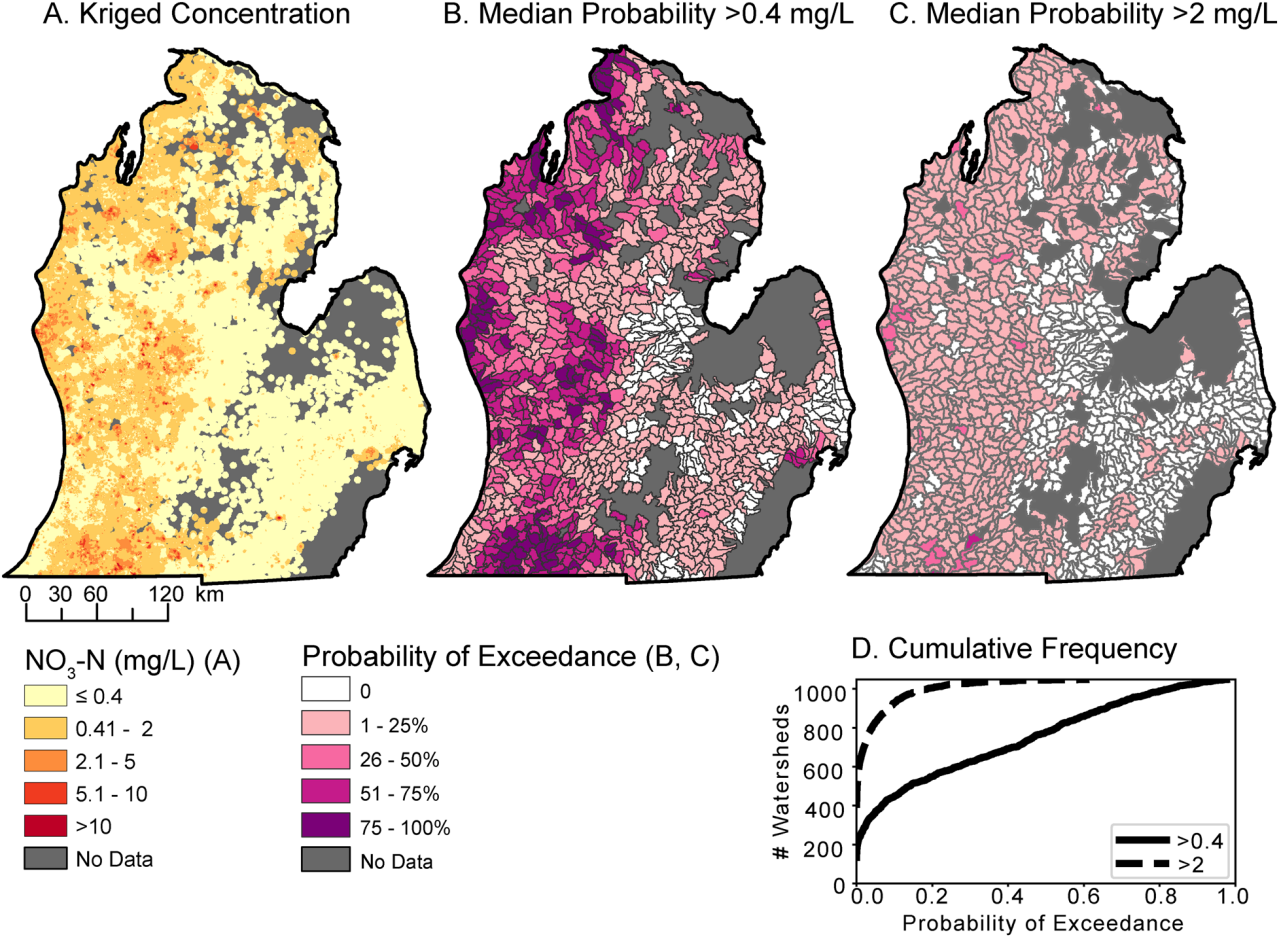
Nitrate estimates for the quaternary aquifer based on samples from 2006 to 2015: (a) Predicted NO_3_‐N concentration clipped to areas of confidence at 250 m resolution, (b) median probability of exceeding 0.4 mg/L NO_3_‐N at HUC12 watershed level, which is the most common detection limit in this region during this sampling period. (c) Median probability of exceeding 2 mg/L NO_3_‐N at HUC12 watershed level. (d) Cumulative frequency of watersheds at both exceedance levels. Note: Low median probabilities can be of significant consequence at local scales.

We were able to gather chemistry data from, and geolocate, 76,724 drinking water wells sampled from 2006 to 2015. These observed concentrations can be viewed as a map showing the well locations and geographic variability for each aquifer (Figure 2). A majority (75%) of the drinking water wells in the study area harvest groundwater stored in the Quaternary‐age aquifer system, although there are areas that rely on the Bedrock aquifer system to access drinking water. Quaternary aquifer wells are most densely located in the western and north central areas of the state, as well as a strip in the eastern portion, directly outside Detroit (Figures 2, 3). These areas correspond to glacial sediment features greater than 50 m thick (Soller & Garrity, 2018).

Groundwater nitrate concentrations across the study area range from non‐detectable (detect limits ranged 0.01–1 mg/L NO^3^‐N) to 59.1 mg/L NO_3_‐N. Background concentrations of nitrate in Michigan range from 0 to 0.27, meaning even non‐detectable samples in this study may be derived from anthropogenic sources (Dumouchelle et al., 1987). The western portion of the state consistently had areas of detectable nitrate concentrations, including in the more sparsely populated northwestern region (Figures 1 and 2), with the highest concentrations found north of Grand Rapids (Figure 2a1) and south of Kalamazoo (Figure 2a2), both corresponding with intensive agricultural areas. Across the study area, 26% of Quaternary wells have detectable nitrate (≥0.4 mg/L NO_3_‐N) and 13% exceed 2 mg/L NO_3_‐N, with the distribution shown in legend of Figure 2a. For both Quaternary and bedrock wells, distributions were highly skewed to non‐detectable concentrations. MCL violations were observed in 1,023 wells (1.8%). Bedrock wells are primarily found in the eastern and south‐central portions of the state, and only 7% of these wells had detectable concentrations (≥0.4 mg/L NO_3_‐N, Figure 2b legend). The remaining analyses focus on Quaternary wells, due to their wide use for drinking water and closer relationship with surficial nitrogen loading processes where humans have more control than in deep bedrock aquifers (Figure 2a).

We used kriging to interpolate the point observations from the 57,469 Quaternary aquifer drinking water wells to create a continuous map layer of groundwater nitrate across the study area (Figure 3). Kriging creates both a smooth surface of predicted concentrations (Figure 3a) and probability layers for exceeding selected thresholds, here >0.4 mg/L NO_3_‐N, which was the mode of the detection limits in the database (Figure S2A in Supporting Information S1), and >2 mg/L NO_3_‐N (Figure S2B in Supporting Information S1). The probability of exceedance can be understood conceptually through the lens of randomly drilling a new groundwater well (although in reality wells and settlements show clustered patterns). A new well drilled within a given watershed would have an expected probability of exceeding the threshold as shown in Figures 3b and 3c. However, as with all probabilities, there may be localized areas within the watershed that will be both more or less likely to exhibit nitrate exceedance than the watershed median.

The interpolated data show a similar pattern of higher nitrate values in the western portion of the region as shown by the direct well observations (Figures 2 and 3). The probability map also highlights the extent of the study area that likely have detectable groundwater nitrate (Figure 3b) and where health risks from elevated nitrate may be a concern (Figure 3c). Much of the western half of the study area has median probabilities of exceeding 0.4 mg/L NO_3_‐N of over 0.5 (50%) (Figure 3b). Specifically, 26% of the watersheds in the study area have a 50% or higher probability of having detectable nitrate in the groundwater (Figure 3d). In contrast, the probability of exceeding 2 mg/L NO_3_‐N is less than 25% for 98% of watersheds (Figure 3d). There are several regions (26 out of 1048 watersheds) in the western LP from North to South that have greater than 25% probability of groundwater nitrate concentrations exceeding 2 mg/L NO_3_‐N (Figure 3c).

### 3.2 Analyzing Relationships Using CART

**Figure 4:**
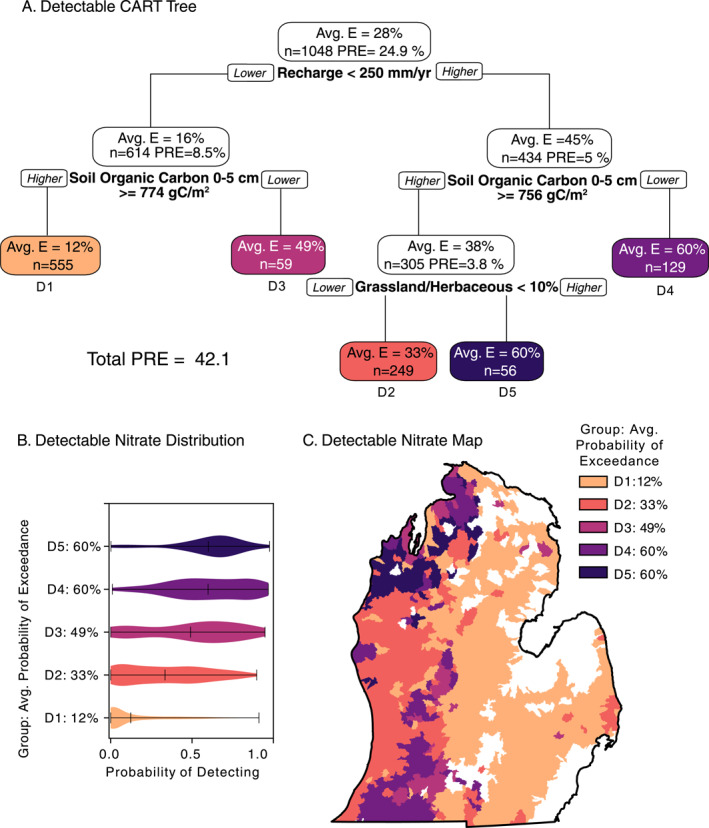
Detectable (>0.4 mg/L NO_3_‐N) nitrate classification and regression tree (CART) results. (a) Decision tree produced by CART for detectable nitrate (>0.4 mg/L NO_3_‐N). *Avg. E*. refers to the average exceedance probability in the group. Variables and thresholds are shown beneath each node. Final groups are labeled with D1–D5, where D1 is the lowest exceedance probability and D5 is the highest. *n* shows the number of watersheds in each group. Proportional reduction in error (PRE) refers to the variability explained by each group, where each node PRE sums to the Total PRE. (b) Distributions shown in violin plots for each CART group. (c) Watershed‐level map colored by CART group. Note: Low median probabilities can be of significant consequence at local scales.

**Figure 5:**
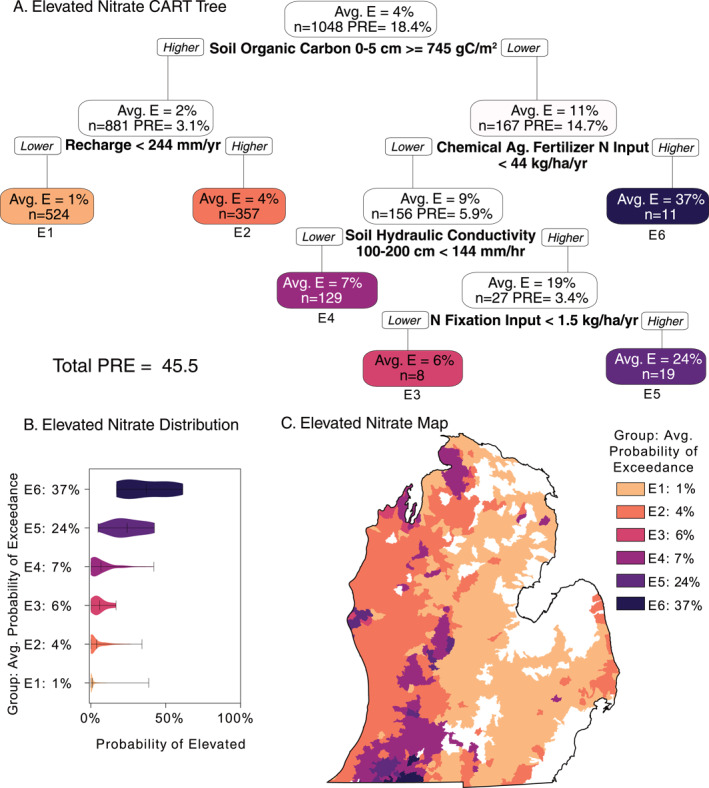
Elevated nitrate (>2 mg/L NO_3_‐N) classification and regression tree (CART) results. (a) Decision tree produced by CART for median watershed nitrate >2 mg/L NO_3_‐N. *Avg. E*. refers to the average exceedance probability in the group. Variables and thresholds are shown beneath each node. Final groups are labeled with E1–E6, where E1 is the lowest exceedance probability and E6 is the highest. *N* shows the number of watersheds in each group. Proportional reduction in error (PRE) refers to the variability explained by each group, where each node PRE sums to the Total PRE. (b) Distributions shown in violin plots for each CART group. (c) Watershed‐level map colored by CART group. Note: low median probabilities can be of significant consequence at local scales.

We used CART analyses to improve the understanding of processes driving patterns in groundwater nitrate concentrations. Results are shown in a “tree” created for each probability threshold (Figures 4 and 5a). For each “node” along the tree we report the average watershed probability of exceedance (*Avg. E*), number of watersheds (*n*), and the proportion reduction of error (PRE). The PRE in each node sums to the CART's total PRE, or ability to explain variability in the data set. Each node is then divided by a “split” condition for a single driver variable and threshold value, which are reported at each split. These splits create two new nodes until the group cannot be split further. These “terminal nodes” are the final CART groups. We used color to denote the rank (i.e., relative probability) among the terminal nodes, ranging from low to high probability (yellow to dark purple, respectively). We used these same colors corresponding to CART terminal node groups in visualizations of distributions and maps. Violin plots (Figures 4b and 5b) are a box and whisker plot overlain with a density plot representing the number of watersheds at each probability of exceedance along the *x*‐axis (Hintze & Nelson, 1998; matplotlib version 3.1.1). These panels provide a nuanced view of our CART results through statistical and spatial lenses.

#### 3.2.1 Probability of Nitrate Presence

Our CART analysis of the probability of detecting nitrate in groundwater (>0.4 mg/L) explained 42% of variability, with just four splits and five terminal groups. Watersheds were first split based on median annual recharge (mm/yr), which is how much water percolates through the subsurface to reach groundwater. Watersheds were split on recharge of 250 mm/yr, explaining 25% of variability within the data set. Roughly 41% of watersheds had recharge greater than this split. Regardless of recharge, the next split was near‐surface SOC (at 0–5 cm depth below the surface). Here, lower recharge watersheds were split at 774 g C/m^2^ and higher recharge watersheds were split at 756 g C/m^2^. Both of these split values are relatively low, as approximately 82% of watersheds had higher average SOC than the average of these split values. On both sides of the tree, lower SOC split out watersheds with some of the highest probabilities of groundwater nitrate detection (49% D3, 60% D4). Watersheds with lower recharge and higher SOC had the lowest probability of groundwater nitrate detection (12%, D1). High recharge and high SOC watersheds on the right portion of the tree were further split by grassland land use/cover, where watersheds with more grassland also had very high probabilities of nitrate detection (60%, D5).

The violin plots (Figure 4b) show the distribution of data in each terminal node. Notably, the extreme groups (D5 and D1, respectively) have the tightest distribution, indicating that the watersheds within those groups have similar probabilities. Alternatively, the two highest groups (D5 and D4) have the same median values (60%) but the distributions are very different. Though the median probability of nitrate detection in these watersheds is 60%, those in group D4 have a more uniform distribution across the full range of probabilities while watersheds in group D5 are clustered around the median value.

Given that recharge is the first split in the CART analysis, it is not surprising that we see the geographic distribution of the five CART groups follow a similar East‐West divide that we describe previously in Figure 3 as being possibly driven by distribution of recharge in the region (Figure 1e). The eastern portion of the state is dominated by watersheds in CART group D1, with lower recharge and higher SOC (Figure 1d). A few areas in the east belong to other CART groups (primarily D2 and D3). Higher nitrate probability clusters are found in the northwestern LP and southwestern LP, classified in groups D3, D4, and D5. D4 and D5, the two highest groups with 60% probability of exceedance, are differentiated spatially (D4: SW; D5: NW). The North‐South D4 cluster stretching from the SW to South‐central corresponds to a band of low SOC (Figure 1d) and significant industrial agriculture (Figure 1d).

#### 3.2.2 Probability of Exceeding 2 mg/L NO_3_‐N

The relationship between the probability of nitrate within a watershed exceeding 2 mg/L NO_3_‐N (where the potential for health concerns rises) and the landscape and geologic properties possibly driving those differences is shown in Figure 5. This CART analysis explained 45% of the variability in these watersheds with six terminal groups. The first split was on SOC at 0–5 cm depth and alone explained 18% of the variability in the data set. This SOC value here (745 mg C/L) is consistent with the range of values in the detectable CART analysis (Figure 4a; SOC splits = 756 and 774). Where SOC was higher (*left side of* Figure 5c), recharge of 244 mm/yr distinguished the two lowest probability groups (E1, E2), a bit less than the 250 mg/L NO_3_‐N observed in Figure 4a. Within the 16% of lower SOC watersheds, CART separated the remaining groups with chemical agricultural fertilizer inputs, soil hydraulic conductivity, and nitrogen fixation from legumes. Notably, chemical agricultural fertilizer inputs >44 kg/ha/yr at the watershed level isolated the highest probability group, where approximately 1 in 3 wells could exceed 2 mg/L (E6). Where fertilizer inputs were lower, watersheds split based on soil hydraulic conductivity. Lower soil conductivity, or slower water flow through soil, resulted in another low probability group (E4). A small subsection of 19 watersheds were isolated with higher conductivity. These areas had lower SOC but were less intensively fertilized and had lower conductivity than the highest groups (E5, E6). The last split separated 2.5% of study area watersheds based on landscape nitrogen fixation inputs from legumes. Higher N Fixation is correlated with higher probability (E5), potentially related to corn‐soy rotation agriculture was present within watersheds.

Distributions shown in violin plots for watershed probability >2 mg/L NO_3_‐N (Figure 5b) generally had less variation than detectable groups (Figure 4b) as evidenced by distributions with rounded bumps and shorter lines. Groups E5 and E6 have significantly elevated probability compared to the remaining lower groups whose central tendencies clustered from 0% to 10%. Group E4 showed the most variability in group membership, as its values were primarily centered around 5% probability, but its 75th percentile whisker extended to 50% probability. Groups E1 and E2, where the majority of watersheds are classified due to their higher SOC, were both centered around 0%–5% probability, but did include some outliers that extended whiskers to ∼45% probability. Generally, these more concise violin plots for elevated nitrate suggest stronger in‐group similarity than for detectable nitrate.

The spatial distribution for >2 mg/L (Figure 5c) shares some similarities with detectable nitrate, but isolates smaller clusters of watersheds with elevated nitrate probabilities and dampens the dominance of an east‐west gradient. Groups E1 and E2, where SOC is higher, exhibit an East‐West gradient related to differences in recharge. The smallest, highest probability groups (E5 and E6) are mainly in clusters in the southwest portion of the state. Group E6, the highest probability group, with only 11 watersheds are all in St. Joseph county, Michigan. This region is characterized by intensive row crop agriculture. The second highest probability group (E5) of 19 watersheds, is more dispersed. It includes cluster neighboring E6, but also appears in the west‐central regions along the coast and further inland. Areas in the the northwestern LP that were classified as high probability of detecting nitrate (Figure 4c, D6) were grouped within the larger, low probability E1 and E2 groups in the >2 mg/L NO_3_‐N CART (Figure 5c, E1 and E2).

## 4 Discussion

This work synthesizes a large database of nitrate concentrations in 76,724 drinking water wells across Michigan's Lower Peninsula, interpolating this point data to cover the majority of watersheds (79%) with continuous estimates of groundwater nitrate concentrations, and contextualizes this in a human health and land management perspective. There is growing concern among health scientists that chronic exposure to levels of nitrate below the current health standard of 10 mg/L NO_3_‐N, even as low as ∼2 mg/L NO_3_‐N, are linked to multiple cancers, adverse birth outcomes, and other negative health problems (Ward et al., 2005, 2018). We provide maps of nitrate levels in our study region as: observed concentration points (Figure 2), kriged concentrations (Figure 3a), kriged probabilities within watersheds (Figures 3b and 3c), and membership of watersheds to CART groups (Figures 4 and 5). These maps provide different views of geographical distribution of groundwater nitrate across our study region. The Kriged concentrations and probability maps are derived from observed data. The maps of CART groups provide a synthetic view that identifies watersheds where the probability of nitrate concentrations meet specific criteria (>0.4 and >2 mg/L) with the landscape characteristics that appear to be driving those spatial patterns. The most important distinction between observational maps (Figures 2 and 3) and CART maps (Figures 4 and 5) is that observational maps can be used to identify areas of risk whereas CART maps group watersheds by similar characteristics that are correlated with risk. CART seeks to explain patterns, but results in groups that may have a wide distribution of concentrations given the small number of terminal groups. For decision making purposes observed data are preferred, but the CART‐identified landscape characteristics can help identify areas that are either at risk or could move into a higher risk level.

We found that a majority of drinking water wells (79%) in both Quaternary‐age and bedrock aquifers had levels of nitrate below the data set's typical analytical detection limit of 0.4 mg/L NO_3_‐N (Figures 2a and 2b). However, there were 11% of wells with concentrations ≥2 mg/L NO_3_‐N, where health concerns may rise (Temkin et al., 2019; Ward et al., 2018). Interpolating these point observations to create a smooth surface map of groundwater nitrate levels (Figure 3) allowed us to summarize results across watersheds, which is an aggregation scale that allows us to analyze relationships between landscape characteristics and groundwater concentrations, and spans the hydrologic pathways controlling groundwater nitrate concentrations.

We found that geologic characteristics associated with transport mechanisms (i.e., aquifer recharge, soil hydraulic conductivity), and landscape features commonly related to agriculture (i.e., SOC, nitrogen inputs from chemical fertilizers and fixation) have important relationships to groundwater nitrate concentration (Figures 4 and 5). The combinations of interpolation and watershed‐based aggregation shown here better represent the distribution of concentrations across the landscape, and summarizes landscape characteristics affecting groundwater concentrations. These landscape patterns are more appropriate to inform broader management decisions. Aside from the east‐west gradient in risk noted above in Results, other prominent patterns in both the probability of detection and elevated levels (Figures 3 and 4) highlight areas of intensive row crop agriculture in the southwest, a mix of agriculture, forest and population centers in the west‐central, and agricultural areas dominated by fruit crops in the northwest. We further evaluate the landscape factors related to these patterns in the discussion of CART results below.

### 4.1 Interpreting Groundwater Nitrate Concentrations and Risk

Our focus was to identify landscape characteristics that are correlated to risk of detection and elevated groundwater nitrate. Here, CART analysis of groundwater nitrate concentrations in small watersheds of Michigan's lower peninsula, a midwestern glacial landscape, identified aquifer recharge, SOC, agricultural nitrogen sources, and soil drainage properties (hydraulic conductivity) as key factors. This is generally consistent with other decision‐tree‐based studies throughout the United States that find variables related to sources, soil properties, and recharge as important factors (Burow et al., 2010; Messier et al., 2019; Pennino et al., 2020; Ransom et al., 2017; Tesoriero et al., 2017; Uddameri et al., 2020). Burow et al. (2010) summarizes nitrate levels across the continental United States (CONUS) as highest in shallow groundwater beneath agricultural land composed of well‐drained soil and oxic geochemical conditions, a useful description of the variety of driving components.

**Figure 6:**
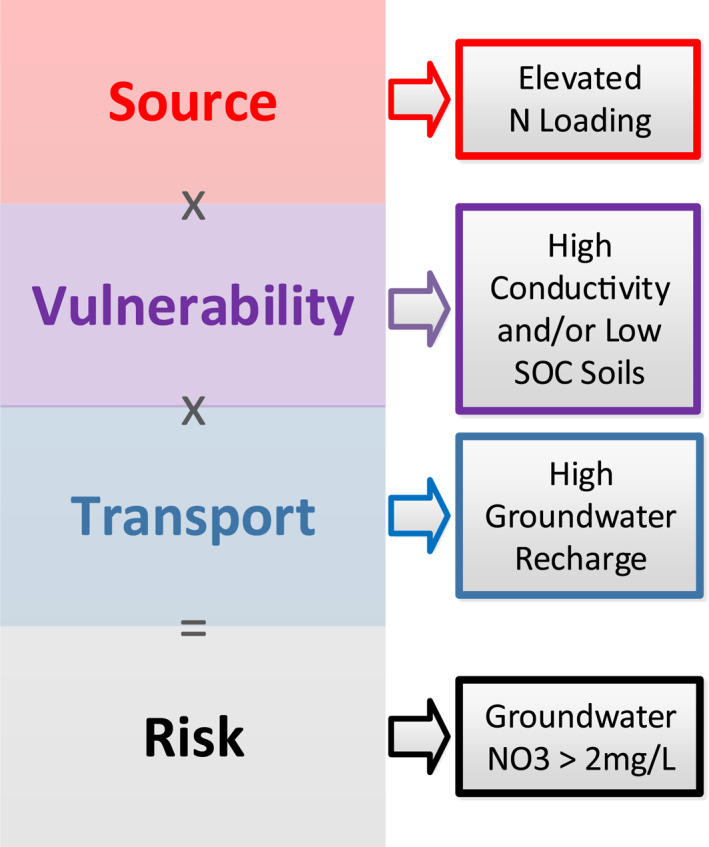
Risk model for high groundwater nitrate, along with associated landscape characteristics identified by our classification and regression tree analyses.

A general conceptual understanding emerges from the literature, supported by this study: elevated groundwater nitrate concentration requires a nitrogen source, vulnerable soil and aquifer properties (including physical and geochemical conditions), and sufficient transport mechanisms (Burow et al., 2010; Hansen et al., 2016; Liggett & Talwar, 2009; Nolan & Hitt, 2006). In our analysis, these all emerge as correlated factors: aquifer recharge (transport), fertilizer and fixation inputs (source), soil conductivity and SOC (vulnerability). We combine these factors into a conceptual model of groundwater nitrate risk, where risk is described via a combination of nitrate source, vulnerability, and transport factors (Figure 6).

#### 4.1.1 Source via Industrial Agriculture

Nitrogen sources, described either by discrete nitrogen inputs or agricultural land use, are identified as key drivers at multiple scales: CONUS (Burow et al., 2010; Nolan & Hitt, 2006; Pennino et al., 2020), Northern Wisconsin (Tesoriero et al., 2017), California's Central Valley (Nolan et al., 2014, 2015), and North Carolina (Messier et al., 2019). Our elevated nitrate risk CART outputs show that landscape nitrogen inputs from SENSEmap (Hamlin et al., 2020a, 2020b) better describe nitrate concentrations than agricultural land use. Neither SENSEmap nitrogen nor agricultural land use are strongly correlated with elevated nitrate via simple correlation (both *r* = 0.06). Nevertheless, the combination of low SOC, high chemical agricultural fertilizers, conductive soils, and high N fixation (a signal of soy/corn rotational cropping) effectively separate the highest risk watersheds from all others (Figure 5). These subtleties highlight the utility of considering both soil properties and individual nitrogen sources, as agricultural land use encompasses many crops, practices, and landscapes. Note that the thresholds identified here in our CARTs are watershed averages, including both agricultural and non‐agricultural areas within the watersheds. Local N input rates can be much greater.

Notably our CART for probability of nitrate detection does not include nitrogen sources, instead only landscape factors such as groundwater recharge and SOC emerge as correlated variables. This is likely due to the ubiquity of nitrogen inputs from atmospheric deposition. Our SENSEmap inputs, shown in Figure 1f, make this clear. Fully 40% of the landscape across Michigan's Lower Peninsula has between 8 and 11 kg/ha/yr of N inputs, and another 20% only slightly less than 8 kg/ha/yr. While some background level of atmospheric deposition of N is due to natural processes alone (such as lightning strikes and atmospheric chemistry), current deposition over the study region is likely 2–3 times greater than prior to 1850 (Bala et al., 2013; Kanakidou et al., 2016). As a result, our CART for likelihood of detection does not find N source as a significant variable because there is enough of an N source everywhere. Those watersheds with lower probability of exceeding the detection threshold (0.4 mg/L NO_3_‐N) have lower vulnerability and transport characteristics, as discussed below.

#### 4.1.2 Vulnerability via Soils

Our analyses identified two soil characteristics that describe the vulnerability of an area to contamination from N sources: SOC and soil hydraulic conductivity. Well drained soil, closely related to hydraulic conductivity used here, has also been extensively identified elsewhere as an important descriptor of groundwater nitrate contamination risk (Burow et al., 2010; Messier at al., 2019; Nolan & Hitt, 2006; Tesoriero et al., 2017). SOC is a complex soil property dictated by geologic, climatologic, and biological processes along with a long history of land use disturbance and agricultural management. The SOC thresholds identified in both our detection and elevated nitrate CARTs effectively separates the bottom 20% of SOC levels in the study area's watersheds. While being a signature of geography and agricultural management in our study area, SOC also describes the complex microbial activity in soil. Soils depleted of or otherwise low in carbon may lack communities of microbes that use and retain nitrogen in the soil, resulting in more nitrate available for leaching. SOC may be acting as a proxy for the combination of management, physical soil properties, and geochemical conditions including both biological activity and redox conditions. The simple correlation between SOC and elevated nitrate exceedance is weakly negative (*r* = −0.18), but our analysis here shows a strong threshold response. Only those soils within the bottom 20% of SOC levels (generally <745–775 gC/m^2^ in the top 5 cm of soil) had higher probabilities of detection or elevated groundwater nitrate. Soil organic matter, a similar variable not quantified in this study, was also identified as an important variable correlated to agricultural activity and clay content by Uddameri et al. (2020).

Our CART also reveals that soils with both high hydraulic conductivity (i.e., which allows rapid movement through the soil) and high nitrogen fixation from legumes (indicative of soy/corn rotation) have high risk of elevated nitrate levels. The map of locations where this group (E5) occurs (Figure 5c) shows two clusters that are areas of mixed agricultural and forested watersheds (Figure 1b). Thus these are areas where locally intensive agriculture is intermixed with forested areas, atop vulnerable low SOC and high conductivity soils.

#### 4.1.3 Transport via Recharge

Our results show that the ability to *transport* nitrate from the surface through the subsurface is a strong control on whether an aquifer's wells will have detectable nitrate, and a weak control on elevated nitrate levels. This is primarily due to the high mobility of nitrate in water. We would expect that well depth would feature importantly, as seen in a number of studies (Burow et al., 2010; Nolan et al., 2014; Tesoriero et al., 2017; Wheeler et al., 2015). However, in our analysis well depth was not a strong descriptor. This may be due to local practices and regulations regarding well depths that preclude shallow wells at the highest risk. Groundwater recharge is a complex variable that encompasses multiple processes, including precipitation, land cover, and soil properties (Healy, 2010). Recharge was also identified as an important variable in Northern Wisconsin (Tesoriero et al., 2017), a similar study area located near Michigan using similar analyses.

In continental US level studies, a key hydrologic transport variable was irrigation:precipitation ratios, which describes some of the highest nitrate concentration regions in the arid and semi‐arid West (Nolan & Hitt, 2006; Pennino et al., 2020). Although Michigan is a humid region, irrigation is used extensively in southwest LP (Morenz et al., 2005) where we found nitrate levels to be the highest, but due to low accuracy in mapping irrigated areas (Xu et al., 2019) we did not include an irrigation variable. A variable describing irrigation‐driven recharge may improve our CART and highlight another avenue in which agricultural management affects nitrate.

Our results stress the importance of geologic and climatic conditions for groundwater nitrate concentrations. Notably, precipitation regimes will likely vary under climate change (IPCC, 2021), meaning the precipitation driver in recharge may change the transport of nitrate to groundwater. Climate change is projected to increase the frequency of intense, but sporadic, precipitation events, which may create pulses of higher nitrogen loading to groundwater and spikes in drinking water (Bowles et al., 2018; Congreves et al., 2016). These near‐surface changes will then propagate through groundwater, subject in some cases to long travel times that generate a legacy of prior land use (Pijanowski et al., 2007; Martin et al., 2011, 2017).

### 4.2 Limitations and Bias of Study

Sources of uncertainty include well nitrate measurements, kriging methods, modeled inputs, CART interpretation, and variables not included in the current analysis. These sources of uncertainty also provide avenues of future work and improvements to this study. The nitrate data set that drives this analysis is limited by non‐random spatial sampling (e.g., samples are only collected where humans are settled, use wells, and at depths the wells were screened) and variable physical conditions for samples (e.g., water table elevation, season, well depth). This non‐random spatial sampling can produce inconsistencies if results are interpreted for largely unsettled areas; for this reason we limited our analysis to sufficiently sampled watersheds (>66% confidently estimated interpolated area, see Section 2). Although there is considerable variability in physical conditions when sampling, this is likely dampened in the interpolation and analysis from a large number of samples under different environments and the 10‐year aggregation period. Future analyses may subset well samples into more specific groups representing similar seasons and well depths.

This study provides a conservative estimate of nitrate concentrations and risk, as the more numerous low concentrations dampen the effect of elevated concentrations in EBK and CART. Although a powerful tool to interpolate based on spatial autocorrelation, kriging uses a built‐in error term that can result in differences between observed and estimated points. Because households are often established in areas of development interest (e.g., lakes) or proximity to roads, this creates a spatial bias that then propagates to well water samples. Due to this sample bias for settled areas, samples are often clustered and may have a wide range of concentrations across a relatively small area. Additionally, wells with high nitrate concentrations may be abandoned for new wells or for alternative drinking water sources (e.g., bottled water, city water), resulting in a dominance of low nitrate wells in the data set. Therefore, kriging produces a conservative estimate of mean watershed‐level nitrate concentrations when compared to the sample mean (Figure S3 in Supporting Information S1). This conservative estimate is further transferred to the watershed‐summarized exceedance probability used in CART, meaning exceedance probabilities are also biased to lower nitrate concentrations. This can also cause difficulty in our CART's ability to create homogenous groups since the number of target watersheds are outnumbered and can in some cases be considered a rare event (DeSimone & Ransom, 2021; Krawczyk, 2016). Each of these complexities add levels of conservatism such that our interpolation and CART results should be interpreted as minimum expectations.

The chosen analysis method, CART, brings its own limitations and considerations. Conclusions drawn from CART analysis are limited by the geographic scope of the data set and correlates selected for the analysis. Since the analyzed CARTs explain approximately 45% of variation, other important driver variables may not be present. Less aggressive “pruning” rules (e.g., allowing for more groups) would increase the variation explained, but decrease the transferability of results by allowing the decision tree to select non‐significant random variability for splits. The variables included largely focus on surface processes when more deep subsurface variables may be needed to explain the patterns. This bias in surface‐focused variables is due to the relative difficulty in obtaining and modeling subsurface data (e.g., aquifer recharge, flowpaths, geologic properties, geochemical and redox conditions) compared to surface and near‐surface processes that are more accessible to study, although future studies will utilize recently released subsurface datasets (Erickson et al., 2021; Starn et al., 2021; Zell & Sanford, 2020). CART's relatively simple tree‐based output is supplemented by detailed results that were analyzed by the authors but not presented here. Specifically, for every split, the *rpart* CART algorithm used here presents alternate (though lower performing) possible splitting variables and thresholds. Future work may use more complex tree‐based methods (e.g., boosted regression trees, random forest) that may provide additional information, and may be able to predict nitrate conditions in unsampled regions and make use of the data collected in earlier decades. We believe CART offers advantages in this study compared to other methods for more clear interpretation and categorizing of watersheds to understand driving processes.

## 5 Conclusions

Overall, the vast majority of the LP of Michigan has generally low nitrate levels, but we encourage careful management of vulnerable geologic areas characterized by well drained soil, high recharge, and low SOC. By focusing on recent and lower nitrate concentrations, we provide a preemptive look at where risks may be elevated in the future, encouraging management changes and precaution today. Beyond the possibility of nitrate concentrations rising above policy‐identified action levels (i.e., 5–10 mg/L NO_3_‐N), there is substantive concern of health risks from prolonged exposure to elevated concentrations above 2 mg/L NO_3_‐N. In Michigan, wells in 11% of watersheds have a greater than 10% probability of exceeding this nitrate threshold today, with risks that may be changing substantially in the future. While these seem like low numbers, elevated drinking water nitrate for 1 in 10 households in a watershed is a substantial concern. Knowing where risk is elevated due to source, vulnerability, and transport factors can help managers protect current water resources and assure clean drinking water for new development.

Although humans have control over the nitrogen sources applied, these geologic and climatic vulnerability drivers make today's management decisions in these areas critical. With this data set, future work can identify geographic areas where these risk factors can be related to epidemiological datasets of health outcomes. Such studies would add to the growing body of literature on adverse health effects from nitrate and could help identify regional drivers of elevated nitrate concentration. As this body of literature strengthens, pressure to lower the nitrate MCL at the federal or state levels may increase. In Michigan, there is already regulation to conduct further suitability analysis for new developments where samples are greater than 5 mg/L, which is half the MCL (MI R 562.414).

To our knowledge, this is the first public visualization of this massive and important data set. This unprecedented amount of data is invaluable to analyze groundwater nitrate, and its relationships to both the landscape and the people relying on those groundwater resources. However, this data set was difficult to utilize despite consistent collection since the 1980s. Due to privacy issues, it could only be obtained via FOIA and was not prepared for modern GIS or data science procedures. Significant work went into obtaining, spatially referencing, and assuring quality of measurements to allow the data to be used for scientific study. We hope that regulatory agencies will continue to share data and encourage broader use for scientists who can help address environmental hazards.

This study utilizes an extensive database including 121,492 drinking water nitrate well measurements across Michigan's Lower Peninsula (2006–2015) in tandem with a suite of variables to identify drivers of groundwater nitrate concentrations using CART analysis. This analysis was consistent with conceptual models about groundwater nitrate behavior, showing higher concentrations require a nitrogen source, vulnerable soil/geologic characteristics, and sufficient transport mechanisms. These were identified as correlates to concentrations in the form of nitrogen inputs from agricultural fertilizer and fixation, soil properties of conductivity and organic carbon content, and aquifer recharge. This extensive data set can be used in future studies linking negative health outcomes to drinking water nitrate intake and identify populations at risk given landscape features.

## Conflict of Interest

The authors declare no conflicts of interest relevant to this study.

## Supporting information

Supporting Information S1

Table S1

## Data Availability

Kriging and CART nitrate data can be accessed at Hydroshare (https://doi.org/10.4211/hs.28d844de3ea0494e8c046248a6b9001a) for any use. The authors are not able to share individual well data obtained from the Michigan Department of Environment, Great Lakes, and Energy. Data available at: Hamlin et al. (2021), Michigan Lower Peninsula Groundwater Nitrate Data set, HydroShare, https://doi.org/10.4211/hs.28d844de3ea0494e8c046248a6b9001a.

## References

[gh2321-bib-0001] Aller, L. (1987). DRASTIC: A standardized system for evaluating ground water pollution potential using hydrogeologic settings. Robert S. Kerr Environmental Research Laboratory, Office of Research and Development, U. S. Environmental Protection Agency.

[gh2321-bib-0002] Ascott, M. J. , Gooddy, D. C. , Fenton, O. , Vero, S. , Ward, R. S. , Basu, N. B. , et al. (2021). The need to integrate legacy nitrogen storage dynamics and time lags into policy and practice. The Science of the Total Environment, 781, 146698. 10.1016/j.scitotenv.2021.146698 33794450

[gh2321-bib-0003] Bala, G. , Devaraju, N. , Chaturvedi, R. K. , Caldeira, K. , & Nemani, R. (2013). Nitrogen deposition: How important is it for global terrestrial carbon uptake? Biogeosciences, 10(11), 7147–7160. 10.5194/bg-10-7147-2013

[gh2321-bib-0004] Beasley, D. B. , Huggins, L. F. , & Monke, A. (1980). ANSWERS: A model for watershed planning. Transactions of the ASAE, 23(4), 938–944. 10.13031/2013.34692

[gh2321-bib-0005] Böhlke, J.‐K. (2002). Groundwater recharge and agricultural contamination. Hydrogeology Journal, 10(1), 153–179. 10.1007/s10040-001-0183-3

[gh2321-bib-0006] Bowles, T. M. , Atallah, S. S. , Campbell, E. E. , Gaudin, A. C. M. , Wieder, W. R. , & Grandy, A. S. (2018). Addressing agricultural nitrogen losses in a changing climate. Nature Sustainability, 1(8), 399–408. 10.1038/s41893-018-0106-0

[gh2321-bib-0007] Brender, J. D. , Weyer, P. J. , Romitti, P. A. , Mohanty, B. P. , Shinde, M. U. , Vuong, A. M. , et al. (2013). Prenatal nitrate intake from drinking water and selected birth defects in offspring of participants in the national birth defects prevention study. Environmental Health Perspectives, 121(9), 1083–1089. 10.1289/ehp.1206249 23771435 PMC3764078

[gh2321-bib-0008] Burow, K. R. , Nolan, B. T. , Rupert, M. G. , & Dubrovsky, N. M. (2010). Nitrate in groundwater of the United States, 1991−2003. Environmental Science & Technology, 44(13), 4988–4997. 10.1021/es100546y 20540531

[gh2321-bib-0009] Coffman, V. R. , Jensen, A. S. , Trabjerg, B. B. , Pedersen, C. B. , Hansen, B. , Sigsgaard, T. , et al. (2021). Prenatal exposure to nitrate from drinking water and markers of fetal growth restriction: A population‐based study of nearly one million Danish‐born children. Environmental Health Perspectives, 129(2), 027002. 10.1289/EHP7331 PMC7861494 33539179

[gh2321-bib-0010] Comly, H. H. (1945). Cyanosis in infants caused by nitrates in well water. Journal of the American Medical Association, 129(2), 112–116. 10.1001/jama.1945.02860360014004

[gh2321-bib-0011] Congreves, K. A. , Dutta, B. , Grant, B. B. , Smith, W. N. , Desjardins, R. L. , & Wagner‐Riddle, C. (2016). How does climate variability influence nitrogen loss in temperate agroecosystems under contrasting management systems? Agriculture, Ecosystems & Environment, 227, 33–41. 10.1016/j.agee.2016.04.025

[gh2321-bib-0012] De’Ath, G. , & Fabricius, K. E. (2000). Classification and regression trees: A powerful yet simple technique for ecological data analysis. Ecology, 81(11), 3178–3192. 10.1890/0012-9658(2000)081[3178:CARTAP]2.0.CO;2

[gh2321-bib-0013] DeSimone, L. A. , McMahon, P. B. , & Rosen, M. R. (2015). The quality of our Nation’s waters: Water quality in principal aquifers of the United States, 1991‐2010. US Geological Survey. 10.3133/cir1360

[gh2321-bib-0014] DeSimone, L. A. , & Ransom, K. M. (2021). Manganese in the Northern Atlantic Coastal Plain aquifer system, eastern USA‐Modeling regional occurrence with pH, redox, and machine learning. Journal of Hydrology: Regional Studies, 37, 100925. 10.1016/j.ejrh.2021.100925

[gh2321-bib-0015] Dumouchelle, D. H. , Cummings, T. R. , & Klepper, G. R. (1987). Michigan ground‐water quality (Vol. 87, p. 0732). US Geological Survey Open‐File Report. 10.3133/ofr87732

[gh2321-bib-0016] Erickson, M. L. , Elliott, S. M. , Brown, C. J. , Stackelberg, P. E. , Ransom, K. M. , & Reddy, J. E. (2021). Machine learning predicted redox conditions in the glacial aquifer system, northern continental United States. Water Resources Research, 57(4), e2020WR028207. 10.1029/2020wr028207

[gh2321-bib-0017] Espejo‐Herrera, N. , Gràcia‐Lavedan, E. , Boldo, E. , Aragonés, N. , Pérez‐Gómez, B. , Pollán, M. , et al. (2016). Colorectal cancer risk and nitrate exposure through drinking water and diet. International Journal of Cancer, 139(2), 334–346. 10.1002/ijc.30083 26954527

[gh2321-bib-0018] Espejo‐Herrera, N. , Gracia‐Lavedan, E. , Pollan, M. , Aragonés, N. , Boldo, E. , Perez‐Gomez, B. , et al. (2016). Ingested nitrate and breast cancer in the Spanish multicase‐control study on cancer (MCC‐Spain). Environmental Health Perspectives, 124(7), 1042–1049. 10.1289/ehp.1510334 26942716 PMC4937871

[gh2321-bib-0019] ESRI (2020a). ArcGIS Pro Version 2.5 [Software].

[gh2321-bib-0020] ESRI . (2020b). What is Empirical Bayesian kriging? Retrieved from https://pro.arcgis.com/en/pro-app/help/analysis/geostatistical-analyst/what-is-empirical-bayesian-kriging-.htm

[gh2321-bib-0021] Fan, A. M. , & Steinberg, V. E. (1996). Health implications of nitrate and nitrite in drinking water: An update on methemoglobinemia occurrence and reproductive and developmental toxicity. Regulatory Toxicology and Pharmacology, 23(1), 35–43. 10.1006/rtph.1996.0006 8628918

[gh2321-bib-0022] Farrand, W. , & Bell, D. (1982). Quaternary geology. Department of Geological Sciences, Univeristy of Michigan. Geologic Survey Division, MDEQ. Division Geographic Information Services Unit, Resource Mapping and Aerial Photography, MDNR. Retrieved from http://www.dnr.state.mi.us/spatialdatalibrary/metadata/quaternary_geology.htm

[gh2321-bib-0023] Faucett, R. L. , & Miller, H. C. (1946). Methemoglobinemia occurring in infants fed milk diluted with well water of high nitrate content. The Journal of Pediatrics, 29(5), 593–596. 10.1016/S0022-3476(46)80126-4 21002863

[gh2321-bib-0024] Fenton, O. , Mellander, P.‐E. , Daly, K. , Wall, D. P. , Jahangir, M. M. R. , Jordan, P. , et al. (2017). Integrated assessment of agricultural nutrient pressures and legacies in karst landscapes. Agriculture, Ecosystems & Environment, 239, 246–256. 10.1016/j.agee.2017.01.014

[gh2321-bib-0025] Ferrant, M. (1946). Methemoglobinemia: Two cases in newborn infants caused by nitrates in well water. The Journal of Pediatrics, 29(5), 585–592. 10.1016/s0022-3476(46)80125-2 21002862

[gh2321-bib-0026] Galloway, J. N. , Dentener, F. J. , Capone, D. G. , Boyer, E. W. , Howarth, R. W. , Seitzinger, S. P. , et al. (2004). Nitrogen cycles: Past, present, and future. Biogeochemistry, 70(2), 153–226. 10.1007/s10533-004-0370-0

[gh2321-bib-0027] Hamlin, Q. F. , Kendall, A. D. , Martin, S. L. , Whitenack, H. D. , Roush, J. A. , Hannah, B. A. , & Hyndman, D. W. (2020a). Quantifying landscape nutrient inputs with spatially explicit nutrient source estimate maps. Journal of Geophysical Research: Biogeosciences, 125(2), 1–24. 10.1029/2019JG005134

[gh2321-bib-0028] Hamlin, Q. F. , Kendall, A. D. , Martin, S. L. , Whitenack, H. D. , Roush, J. A. , Hannah, B. A. , & Hyndman, D. W. (2020b). SENSEmap‐USGLB: Nitrogen and phosphorus inputs. [Data Set]. Hydroshare. Retrieved from 10.4211/hs.1a116e5460e24177999c7bd6f8292421

[gh2321-bib-0029] Hamlin, Q. F. , Martin, S. , Kendall, A. D. , & Hyndman, D. W. (2021). Michigan lower peninsula groundwater nitrate dataset. [Data Set]. HydroShare. 10.4211/hs.28d844de3ea0494e8c046248a6b9001a

[gh2321-bib-0030] Hansen, B. , Sonnenborg, T. O. , Møller, I. , Bernth, J. D. , Høyer, A.‐S. , Rasmussen, P. , et al. (2016). Nitrate vulnerability assessment of aquifers. Environmental Earth Sciences, 75(12), 1–15. 10.1007/s12665-016-5767-2

[gh2321-bib-0031] Hansen, B. , Thorling, L. , Schullehner, J. , Termansen, M. , & Dalgaard, T. (2017). Groundwater nitrate response to sustainable nitrogen management. Scientific Reports, 7(1), 1–12. 10.1038/s41598-017-07147-2 28819258 PMC5561247

[gh2321-bib-0032] Healy, R. W. (2010). Estimating groundwater recharge. Cambridge university press.

[gh2321-bib-0033] Helsel, D. R. , & Hirsch, R. M. (1992). Statistical methods in water resources (Vol. 49). Elsevier.

[gh2321-bib-0034] Hintze, J. L. , & Nelson, R. D. (1998). Violin plots: A box plot‐density trace synergism. The American Statistician, 52(2), 181–184. 10.1080/00031305.1998.10480559

[gh2321-bib-0035] Holtby, C. E. , Guernsey, J. R. , Allen, A. C. , VanLeeuwen, J. A. , Allen, V. M. , & Gordon, R. J. (2014). A population‐based case‐control study of drinking‐water nitrate and congenital anomalies using geographic information systems (GIS) to develop individual‐level exposure estimates. International Journal of Environmental Research and Public Health, 11(2), 1803–1823. 10.3390/ijerph110201803 24503976 PMC3945569

[gh2321-bib-0036] Holtschlag, D. J. (1997). A generalized estimate of ground‐water‐recharge rates in the Lower Peninsula of Michigan (Vol. 2437). US Department of the Interior, US Geological Survey.

[gh2321-bib-0037] Homer, C. G. , Dewitz, J. A. , Yang, L. , Jin, S. , Danielson, P. , Xian, G. , et al. (2015). Completion of the 2011 National Land Cover Database for the conterminous United States‐Representing a decade of land cover change information. Photogrammetric Engineering & Remote Sensing, 81(5), 345–354. 10.14358/PERS.81.5.345

[gh2321-bib-0038] Hussain, I. , Pilz, J. , & Spoeck, G. (2010). Hierarchical Bayesian space‐time interpolation versus spatio‐temporal BME approach. Advances in Geosciences, 25, 97–102. 10.5194/adgeo-25-97-2010

[gh2321-bib-0039] Hyndman, D. W. , Kendall, A. D. , & Welty, N. R. H. (2007). Evaluating temporal and spatial variations in recharge and streamflow using the integrated landscape hydrology model (ILHM). Geophysical Monograph‐American Geophysical Union, 171, 121–141. 10.1029/171GM11

[gh2321-bib-0040] Inoue‐Choi, M. , Jones, R. R. , Anderson, K. E. , Cantor, K. P. , Cerhan, J. R. , Krasner, S. , et al. (2015). Nitrate and nitrite ingestion and risk of ovarian cancer among postmenopausal women in Iowa. International Journal of Cancer, 137(1), 173–182. 10.1002/ijc.29365 25430487 PMC4405451

[gh2321-bib-0041] IPCC . (2021). Summary for policymakers. In P. Zhai , A. Pirani , S. L. Connors , C. Péan , S. Berger , & N. Caud (Eds.), Climate change 2021: The physical science basis. Contribution of working group I to the sixth assessment report of the intergovernmental panel on climate change [Masson‐Delmotte, V…. Cambridge University Press. In Press.

[gh2321-bib-0042] Johnson, T. D. , Belitz, K. , & Lombard, M. A. (2019). Estimating domestic well locations and populations served in the contiguous US for years 2000 and 2010. The Science of the Total Environment, 687, 1261–1273. 10.1016/j.scitotenv.2019.06.036 31412460

[gh2321-bib-0043] Jones, R. R. , Weyer, P. J. , DellaValle, C. T. , Inoue‐Choi, M. , Anderson, K. E. , Cantor, K. P. , et al. (2016). Nitrate from drinking water and diet and bladder cancer among postmenopausal women in Iowa. Environmental Health Perspectives, 124(11), 1751–1758. 10.1289/EHP191 27258851 PMC5089883

[gh2321-bib-0044] Kanakidou, M. , Myriokefalitakis, S. , Daskalakis, N. , Fanourgakis, G. , Nenes, A. , Baker, A. R. , et al. (2016). Past, present, and future atmospheric nitrogen deposition. Journal of the Atmospheric Sciences, 73(5), 2039–2047. 10.1175/JAS-D-15-0278.1 32747838 PMC7398418

[gh2321-bib-0045] Kendall, A. (2009). Predicting the impacts of land use and climate on regional‐scale hydrologic fluxes. Michigan State University.

[gh2321-bib-0046] Kim, H. , Surdyk, N. , Møller, I. , Graversgaard, M. , Blicher‐Mathiesen, G. , Henriot, A. , et al. (2020). Lag time as an indicator of the link between agricultural pressure and drinking water quality state. Water, 12(9), 2385. 10.3390/w12092385

[gh2321-bib-0047] Krawczyk, B. (2016). Learning from imbalanced data: Open challenges and future directions. Progress in Artificial Intelligence, 5(4), 221–232. 10.1007/s13748-016-0094-0

[gh2321-bib-0048] Liggett, J. E. , & Talwar, S. (2009). Groundwater vulnerability assessments and integrated water resource management. Streamline Watershed Management Bulletin, 13(1), 18–29.

[gh2321-bib-0049] Luscz, E. C. , Kendall, A. D. , & Hyndman, D. W. (2015). High resolution spatially explicit nutrient source models for the Lower Peninsula of Michigan. Journal of Great Lakes Research, 41(2), 618–629. 10.1016/j.jglr.2015.02.004

[gh2321-bib-0050] Luscz, E. C. , Kendall, A. D. , & Hyndman, D. W. (2017). A spatially explicit statistical model to quantify nutrient sources, pathways, and delivery at the regional scale. Biogeochemistry, 133(1), 37–57. 10.1007/s10533-017-0305-1

[gh2321-bib-0051] Machiwal, D. , Jha, M. K. , Singh, V. P. , & Mohan, C. (2018). Assessment and mapping of groundwater vulnerability to pollution: Current status and challenges. Earth‐Science Reviews, 185, 901–927. 10.1016/j.earscirev.2018.08.009

[gh2321-bib-0052] Manassaram, D. M. , Backer, L. C. , & Moll, D. M. (2006). A review of nitrates in drinking water: Maternal exposure and adverse reproductive and developmental outcomes. Environmental Health Perspectives, 114(3), 320–327. 10.1289/ehp.8407 16507452 PMC1392223

[gh2321-bib-0053] Martin, S. L. , Hamlin, Q. F. , Kendall, A. D. , Wan, L. , & Hyndman, D. W. (2021). The land use legacy effect: Looking back to see a path forward to improve management. Environmental Research Letters, 16(3), 35005. 10.1088/1748-9326/abe14c

[gh2321-bib-0054] Martin, S. L. , Hayes, D. B. , Kendall, A. D. , & Hyndman, D. W. (2017). The land‐use legacy effect: Towards a mechanistic understanding of time‐lagged water quality responses to land use/cover. The Science of the Total Environment, 579, 1794–1803. 10.1016/j.scitotenv.2016.11.158 27932215

[gh2321-bib-0055] Martin, S. L. , Hayes, D. B. , Rutledge, D. T. , & Hyndman, D. W. (2011). The land‐use legacy effect: Adding temporal context to lake chemistry. Limnology & Oceanography, 56(6), 2362–2370. 10.4319/lo.2011.56.6.2362

[gh2321-bib-0056] Mattix, K. D. , Winchester, P. D. , & Scherer, L. T. (2007). Incidence of abdominal wall defects is related to surface water atrazine and nitrate levels. Journal of Pediatric Surgery, 42(6), 947–949. 10.1016/j.jpedsurg.2007.01.027 17560200

[gh2321-bib-0057] McElroy, J. A. , Trentham‐Dietz, A. , Gangnon, R. E. , Hampton, J. M. , Bersch, A. J. , Kanarek, M. S. , & Newcomb, P. A. (2008). Nitrogen‐nitrate exposure from drinking water and colorectal cancer risk for rural women in Wisconsin, USA. Journal of Water and Health, 6(3), 399–409. 10.2166/wh.2008.048 19108561

[gh2321-bib-0058] Messier, K. P. , Wheeler, D. C. , Flory, A. R. , Jones, R. R. , Patel, D. , Nolan, B. T. , & Ward, M. H. (2019). Modeling groundwater nitrate exposure in private wells of North Carolina for the Agricultural Health Study. The Science of the Total Environment, 655, 512–519. 10.1016/j.scitotenv.2018.11.022 30476830 PMC6581064

[gh2321-bib-0059] Michigan Compiled Law 324.8713 (MI MCL 324.8713) . Part 87 Groundwater and freshwater protection”. Michigan Natural Resources Act. Accessed online on 23 August 2021. Retrieved from http://www.legislature.mi.gov/documents/mcl/pdf/mcl-act-451-of-1994.pdf

[gh2321-bib-0060] Michigan Rule 562.412 (MI R 562.414) . Part 4 Department of environmental quality on‐site water supply and sewage disposal for land divisions and subdivisions. State of Michigan Administrative Code. Accessed online on 24 August 2021. Retrieved from https://www.michigan.gov/documents/deq/deq-wb-dwehs-ows-subdivision_rules_241122_7.pdf

[gh2321-bib-0061] Michigan Waterwells for WELLOGIC dataset . [Dataset] (2019). Accessed 15 December 2019. Retrieved from https://gis-michigan.opendata.arcgis.com/search?collection=Dataset&q=Wellogic

[gh2321-bib-0062] Morenz, M. L. , Van Til, R. L. , & Luukkonen, C. L. (2005). Water use for irrigation in Michigan, 2001. US Geological Survey. 10.3133/fs20053079

[gh2321-bib-0063] Motevalli, A. , Naghibi, S. A. , Hashemi, H. , Berndtsson, R. , Pradhan, B. , & Gholami, V. (2019). Inverse method using boosted regression tree and k‐nearest neighbor to quantify effects of point and non‐point source nitrate pollution in groundwater. Journal of Cleaner Production, 228, 1248–1263. 10.1016/j.jclepro.2019.04.293

[gh2321-bib-0064] Mueller, B. A. , Nielsen, S. S. , Preston‐Martin, S. , Holly, E. A. , Cordier, S. , Filippini, G. , et al. (2004). Household water source and the risk of childhood brain tumours: Results of the SEARCH international brain tumor study. International Journal of Epidemiology, 33(6), 1209–1216. 10.1093/ije/dyh215 15567873

[gh2321-bib-0065] Nolan, B. T. , Fienen, M. N. , & Lorenz, D. L. (2015). A statistical learning framework for groundwater nitrate models of the Central Valley, California, USA. Journal of Hydrology, 531, 902–911. 10.1016/j.jhydrol.2015.10.025

[gh2321-bib-0066] Nolan, B. T. , Gronberg, J. M. , Faunt, C. C. , Eberts, S. M. , & Belitz, K. (2014). Modeling nitrate at domestic and public‐supply well depths in the Central Valley, California. Environmental Science & Technology, 48(10), 5643–5651. 10.1021/es405452q 24779475

[gh2321-bib-0067] Nolan, B. T. , & Hitt, K. J. (2006). Vulnerability of shallow groundwater and drinking‐water wells to nitrate in the United States. Environmental Science & Technology, 40(24), 7834–7840. 10.1021/es060911u 17256535

[gh2321-bib-0068] Pennino, M. J. , Compton, J. E. , & Leibowitz, S. G. (2017). Trends in drinking water nitrate violations across the United States. Environmental Science & Technology, 51(22), 13450–13460. 10.1021/acs.est.7b04269 29052975 PMC5764095

[gh2321-bib-0069] Pennino, M. J. , Leibowitz, S. G. , Compton, J. E. , Hill, R. A. , & Sabo, R. D. (2020). Patterns and predictions of drinking water nitrate violations across the conterminous United States. The Science of the Total Environment, 722, 137661. 10.1016/j.scitotenv.2020.137661 32192969 PMC8204728

[gh2321-bib-0070] Pijanowski, B. , Ray, D. K. , Kendall, A. D. , Duckles, J. M. , & Hyndman, D. W. (2007). Using backcast land‐use change and groundwater travel‐time models to generate land‐use legacy maps for watershed management. Ecology and Society, 12(2). 10.5751/es-02154-120225

[gh2321-bib-0071] PRISM Climate Group (2012). PRISM. Oregon State University. Retrieved from http://prism.oregonstate.edu/

[gh2321-bib-0072] Ransom, K. M. , Nolan, B. T. , Stackelberg, P. E. , Belitz, K. , & Fram, M. S. (2021). Machine learning predictions of nitrate in groundwater used for drinking supply in the conterminous United States (p. 151065). Science of The Total Environment. 10.1016/j.scitotenv.2021.151065 34673076

[gh2321-bib-0073] Ransom, K. M. , Nolan, B. T. , Traum, J. A. , Faunt, C. C. , Bell, A. M. , Gronberg, J. A. M. , et al. (2017). A hybrid machine learning model to predict and visualize nitrate concentration throughout the Central Valley aquifer, California, USA. The Science of the Total Environment, 601, 1160–1172. 10.1016/j.scitotenv.2017.05.192 28599372

[gh2321-bib-0074] R Core Team (2019). R: A language and environment for statistical computing. [Software]. R Foundation for Statistical Computing. Retrieved from https://r-project.orgversion3.6.0

[gh2321-bib-0075] Rivett, M. O. , Buss, S. R. , Morgan, P. , Smith, J. W. N. , & Bemment, C. D. (2008). Nitrate attenuation in groundwater: A review of biogeochemical controlling processes. Water Research, 42(16), 4215–4232. 10.1016/j.watres.2008.07.020 18721996

[gh2321-bib-0076] Schaap, M. G. , Leij, F. J. , & Van Genuchten, M. T. (2001). Rosetta: A computer program for estimating soil hydraulic parameters wit hierarchical pedotransfer functions. Journal of Hydrology, 25(3–4), 163–176. 10.1061/(ASCE)IR.1943-4774.0000007

[gh2321-bib-0077] Schilling, K. E. , & Jacobson, P. (2010). Groundwater conditions under a reconstructed prairie chronosequence. Agriculture, Ecosystems & Environment, 135(1–2), 81–89. 10.1016/j.agee.2009.08.013

[gh2321-bib-0078] Schullehner, J. , Hansen, B. , Thygesen, M. , Pedersen, C. B. , & Sigsgaard, T. (2018). Nitrate in drinking water and colorectal cancer risk: A nationwide population‐based cohort study. International Journal of Cancer, 143(1), 73–79. 10.1002/ijc.31306 29435982

[gh2321-bib-0079] Sherris, A. R. , Baiocchi, M. , Fendorf, S. , Luby, S. P. , Yang, W. , & Shaw, G. M. (2021). Nitrate in drinking water during pregnancy and spontaneous preterm birth: A retrospective within‐mother analysis in California. Environmental Health Perspectives, 129(5), 057001. 10.1289/EHP8205 PMC8098122 33949893

[gh2321-bib-0080] Soil Survey Staff [Dataset] (2020). Gridded soil Survey geographic (gSSURGO) database for the United States of America and the territories, commonwealths, and island nations served by the USDA NRCS. United States Department of Agriculture, Natural Resources Conservation Service. Available online at https://gdg.sc.egov.usda.gov/

[gh2321-bib-0081] Soller, D. , & Garrity, C. (2018). Quaternary sediment thickness and bedrock topography of the glaciated United States east of the Rocky Mountains. U.S Geological Survey Scientific Investigations Map, 3392. 10.3133/sim3392

[gh2321-bib-0082] Starn, J. J. , Kauffman, L. J. , Carlson, C. S. , Reddy, J. E. , & Fienen, M. N. (2021). Three‐dimensional distribution of groundwater residence time metrics in the glaciated United States using metamodels trained on general numerical simulation models. Water Resources Research, 57(2), e2020WR027335. 10.1029/2020wr027335

[gh2321-bib-0083] Stayner, L. T. , Schullehner, J. , Semark, B. D. , Jensen, A. S. , Trabjerg, B. B. , Pedersen, M. , et al. (2021). Exposure to nitrate from drinking water and the risk of childhood cancer in Denmark. Environment International, 155, 106613. 10.1016/j.envint.2021.106613 33965769

[gh2321-bib-0084] Šimůnek, J. , & van Genuchten, M. T. (2008). Modeling nonequilibrium flow and transport processes using HYDRUS. Vadose Zone Journal, 7(2), 782–797. 10.2136/vzj2007.0074

[gh2321-bib-0085] Temkin, A. , Evans, S. , Manidis, T. , Campbell, C. , & Naidenko, O. V. (2019). Exposure‐based assessment and economic valuation of adverse birth outcomes and cancer risk due to nitrate in United States drinking water. Environmental Research, 176, 108442. 10.1016/j.envres.2019.04.009 31196558

[gh2321-bib-0086] Tesoriero, A. J. , Gronberg, J. A. , Juckem, P. F. , Miller, M. P. , & Austin, B. P. (2017). Predicting redox‐sensitive contaminant concentrations in groundwater using random forest classification. Water Resources Research, 53(8), 7316–7331. 10.1002/2016WR020197

[gh2321-bib-0087] Therneau, T. , Atkinson, B. , & Ripley, B. (2019). rpart 4.1.15. Retrieved from https://cran.r-project.org/package=rpart

[gh2321-bib-0088] Uddameri, V. , Silva, A. L. B. , Singaraju, S. , Mohammadi, G. , & Hernandez, E. A. (2020). Tree‐based modeling methods to predict nitrate exceedances in the Ogallala aquifer in Texas. Water, 12(4), 1023. 10.3390/w12041023

[gh2321-bib-0089] U.S. Census Bureau . (2010). Census of population and housing, population and housing unit counts, CPH‐2‐24. Michigan U.S. Government Printing Office.

[gh2321-bib-0090] USDA National Agricultural Statistics Service Cropland Data Layer . (2010‐2015) [Dataset]. Published crop‐specific data layer [online]. Accessed on 05 May 2016. Retrieved from https://nassgeodata.gmu.edu/CropScape/

[gh2321-bib-0091] USEPA . (2021). National primary drinking water regulations. Accessed on 23 August 2021. Available online https://www.epa.gov/ground-water-and-drinking-water/national-primary-drinking-water-regulations

[gh2321-bib-0092] U.S. Geological Survey (USGS) . (2013). Federal standards and procedures for the national watershed boundary dataset (WBD) (4 ed.): U.S. Geological Survey Techniques and Methods 11–A3 (p. 63). U. S. Department of Agriculture, Natural Resources Conservation Service. Available on the World Wide Web at http://pubs.usgs.gov/tm/tm11a3/

[gh2321-bib-0093] Van Meter, K. J. , & Basu, N. B. (2015). Catchment legacies and time lags: A parsimonious watershed model to predict the effects of legacy storage on nitrogen export. PLoS One, 10(5), e0125971. 10.1371/journal.pone.0125971 25985290 PMC4436186

[gh2321-bib-0094] Vero, S. E. , Basu, N. B. , Van Meter, K. , Richards, K. G. , Mellander, P.‐E. , Healy, M. G. , & Fenton, O. (2018). The environmental status and implications of the nitrate time lag in Europe and North America. Hydrogeology Journal, 26(1), 7–22. 10.1007/s10040-017-1650-9

[gh2321-bib-0095] Vitousek, P. M. , Aber, J. D. , Howarth, R. W. , Likens, G. E. , Matson, P. A. , Schindler, D. W. , et al. (1997). Human alteration of the global nitrogen cycle: Sources and consequences. Ecological Applications. 10.1890/1051-10030761(1997)007[0737:HAOTGN]2.0.CO;2

[gh2321-bib-0096] Voutchkova, D. D. , Schullehner, J. , Rasmussen, P. , & Hansen, B. (2021). A high‐resolution nitrate vulnerability assessment of sandy aquifers (DRASTIC‐N). Journal of Environmental Management, 277, 111330. 10.1016/j.jenvman.2020.111330 32971506

[gh2321-bib-0097] Waller, S. A. , Paul, K. , Peterson, S. E. , & Hitti, J. E. (2010). Agricultural‐related chemical exposures, season of conception, and risk of gastroschisis in Washington State. American Journal of Obstetrics and Gynecology, 202(3), 241‐e1. 10.1016/j.ajog.2010.01.023 20207240

[gh2321-bib-0098] Ward, M. H. , DeKok, T. M. , Levallois, P. , Brender, J. , Gulis, G. , Nolan, B. T. , & VanDerslice, J. (2005). Workgroup report: Drinking‐water nitrate and health—Recent findings and research needs. Environmental Health Perspectives, 113(11), 1607–1614. 10.1289/ehp.8043 16263519 PMC1310926

[gh2321-bib-0099] Ward, M. H. , Jones, R. R. , Brender, J. D. , De Kok, T. M. , Weyer, P. J. , Nolan, B. T. , et al. (2018). Drinking water nitrate and human health: An updated review. International Journal of Environmental Research and Public Health, 15(7), 1557. 10.3390/ijerph15071557 PMC6068531 30041450

[gh2321-bib-0100] Ward, M. H. , Kilfoy, B. A. , Weyer, P. J. , Anderson, K. E. , Folsom, A. R. , & Cerhan, J. R. (2010). Nitrate intake and the risk of thyroid cancer and thyroid disease. Epidemiology (Cambridge, Mass.), 21(3), 389–395. 10.1097/EDE.0b013e3181d6201d PMC2879161 20335813

[gh2321-bib-0101] Weyer, P. J. , Brender, J. D. , Romitti, P. A. , Kantamneni, J. R. , Crawford, D. , Sharkey, J. R. , et al. (2014). Assessing bottled water nitrate concentrations to evaluate total drinking water nitrate exposure and risk of birth defects. Journal of Water and Health, 12(4), 755–762. 10.2166/wh.2014.237 25473985 PMC5072402

[gh2321-bib-0102] Wheeler, D. C. , Nolan, B. T. , Flory, A. R. , DellaValle, C. T. , & Ward, M. H. (2015). Modeling groundwater nitrate concentrations in private wells in Iowa. The Science of the Total Environment, 536, 481–488. 10.1016/j.scitotenv.2015.07.080 26232757 PMC6397646

[gh2321-bib-0103] Wiley, M. J. , Hyndman, D. W. , Pijanowski, B. C. , Kendall, A. D. , Riseng, C. , Rutherford, E. S. , et al. (2010). A multi‐modeling approach to evaluating climate and land use change impacts in a Great Lakes River Basin. In Global change and river ecosystems—Implications for structure, function and ecosystem services (pp. 243–262). Springer. 10.1007/978-94-007-0608-8_17

[gh2321-bib-0104] Winchester, P. D. , Huskins, J. , & Ying, J. (2009). Agrichemicals in surface water and birth defects in the United States. Acta Paediatrica, 98(4), 664–669. 10.1111/j.1651-2227.2008.01207.x 19183116 PMC2667895

[gh2321-bib-0105] World Health Organization . (2017). Guidelines for drinking‐water quality: First addendum to the (4th ed.). 28759192

[gh2321-bib-0106] Xu, T. , Deines, J. M. , Kendall, A. D. , Basso, B. , & Hyndman, D. W. (2019). Addressing challenges for mapping irrigated fields in subhumid temperate regions by integrating remote sensing and hydroclimatic data. Remote Sensing, 11(3), 370. 10.3390/rs11030370\

[gh2321-bib-0107] Zell, W. O. , & Sanford, W. E. (2020). Calibrated simulation of the long‐term average surficial groundwater system and derived spatial distributions of its characteristics for the contiguous United States. Water Resources Research, 56(8), e2019WR026724. 10.1029/2019wr026724

