# Supplementary material for: Examining Relationships Between Groundwater Nitrate Concentrations in Drinking Water and Landscape Characteristics to Understand Health Risks
Source: Geohealth. 2022 May 1;6(5):e2021GH000524. doi: 10.1029/2021GH000524 (PMC9060635; doi:10.1029/2021GH000524)
Supplement: Supplementary file 1 — Supporting Information S1 [file GH2-6-e2021GH000524-s002.pdf]

Geohealth

Supporting Information for

Examining Relationships Between Groundwater Nitrate Concentrations in Drinking Water and Landscape Characteristics to Understand Health Risks

Q. F. Hamlin[1], S. L. Martin[1], A. D. Kendall[1], D. W. Hyndman[1,2]

[1] Department of Earth and Environmental Sciences, Michigan State University, East Lansing, MI, USA

[2] Department Geosciences, School of Natural Sciences and Mathematics, University of Texas at Dallas, Richardson, TX, USA

Contents of this file

Figure S1-S3
Text S1
Table S1

Additional Supporting Information (Files uploaded separately)

Captions for Tables S2

Introduction

Supporting information includes information on kriging methods, outputs from intermediate analysis steps, and a complete table of variables included in CART analysis.

Text S1. Extended Methods: Kriging

Empirical Bayesian Kriging uses a variety of parameters to produce multiple simulations at any prediction point on the kriging surface. The parameters in Table S2 are used in ArcGIS Pro 2.5's Empirical Bayesian Kriging function. Given our parameter choice, for each 250 m cell, EBK simulates 100 semivariogram (*number of simulations*) using the nearest 50 points (*subset size)*. Data points are transformed using a normal scores empirical transformation (*transformation)*. The simulated semivariograms all use the exponential function (*semivariogram type*). After simulating multiple semivariograms iteratively, the cell's value is calculated from 5-12 neighbors (*Min-Max Neighbors*).

Sample semivariograms are shown in FIgure S2 demonstrating three points across the LP. S2A and S2B have a reasonable fit with the exponential shape of points. Blue lines represent additional simulations within the 100. Fig S2A shows that spatial autocorrelation is highest (semivariance lowest on y-axis) at distances < 850 meters and increases until meeting its range (distance where spatial autocorrelation ceases) at 3.5 kilometers. Fig S2A shows some variability in semivariance after the range determined by the model. Fig S2B shows a smoother transition in spatial autocorrelation; however, semivariance values even at low distance (<3.5 km) are significantly increase significantly faster than Fig S2B (semivariance values range from 0 - 9 in Fig S2B compared to 0 - 1.2 in Fig S2A and S2C). Fig S2C shows a cell in the landscape where the semivariogram model shows no relationship between distance and semivariance as semivariance is stable from 0 - 8 km. These semivariograms show just three models across a wide domain but demonstrate the power of EBK to locally represent spatial autocorrelation.

Table S1. Empirical Bayseian Kriging Parameters

**Parameter**	**Value**	**Description**
Cell Size	250 m	Output predicted cells
Subset Size	50	Maximum number of points used in a simulation subset
Number of Simulations	100	Number of semivariograms simulated for each subset
Transformation	Empirical	Normal scores data transformation type
Semivariogram Type	Exponential	Function type used for semivariograms
Min - Max Neighbors	5 - 12	Neighbors used per cell cell calculation

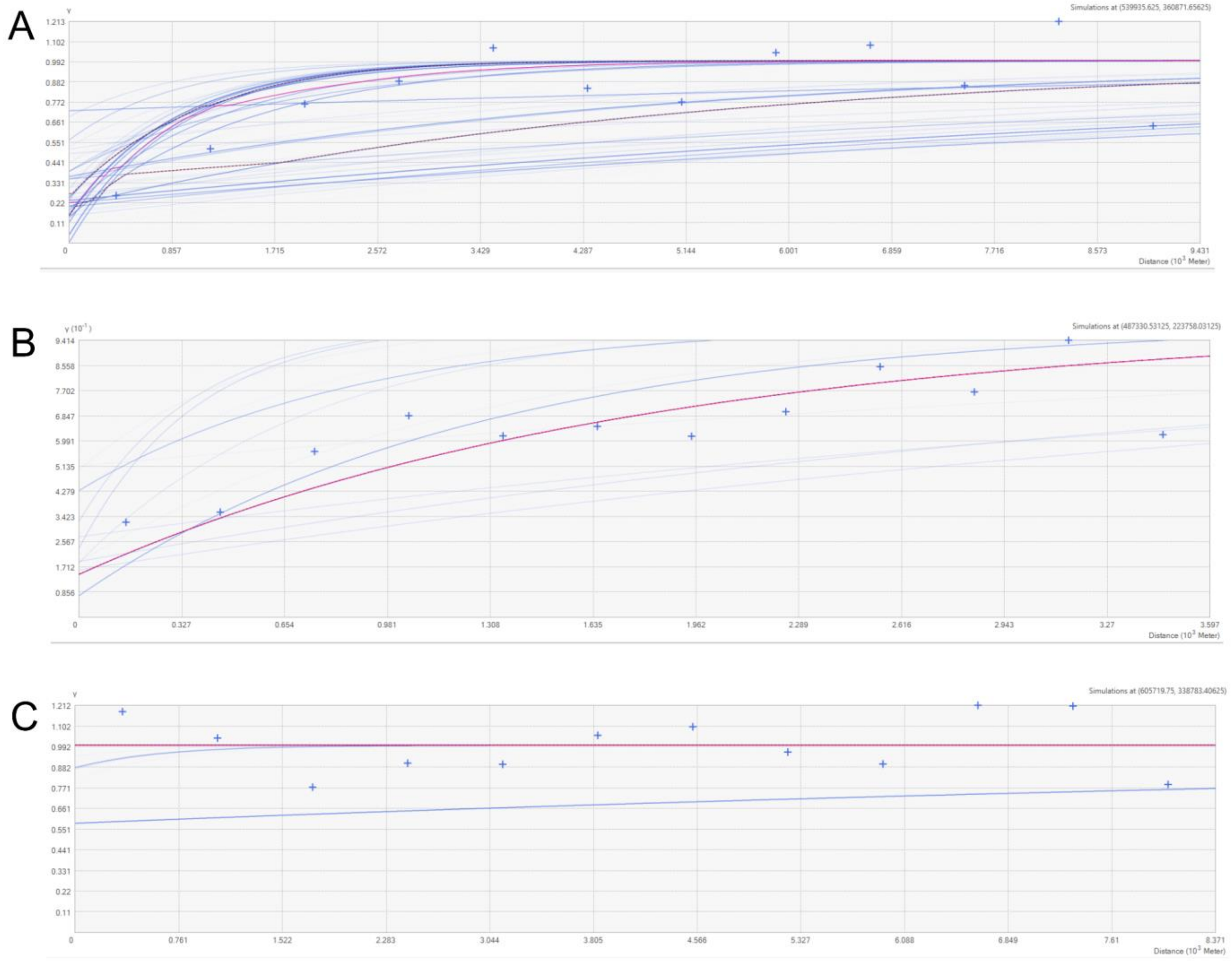

Fig S1. Sample simulated semivariograms using EBK. (A) and (B) show more typical exponential shapes compared to (C) where there is no consistent relationship between spatial autocorrelation, producing a flat semivariogram line. Lines represent multiple simulations using different point subsets, where red is the central curve, dotted red lines represent 25th and 75th percentile, and blue are additional simulations. Crosses represent empirical, or observed, semivariance.

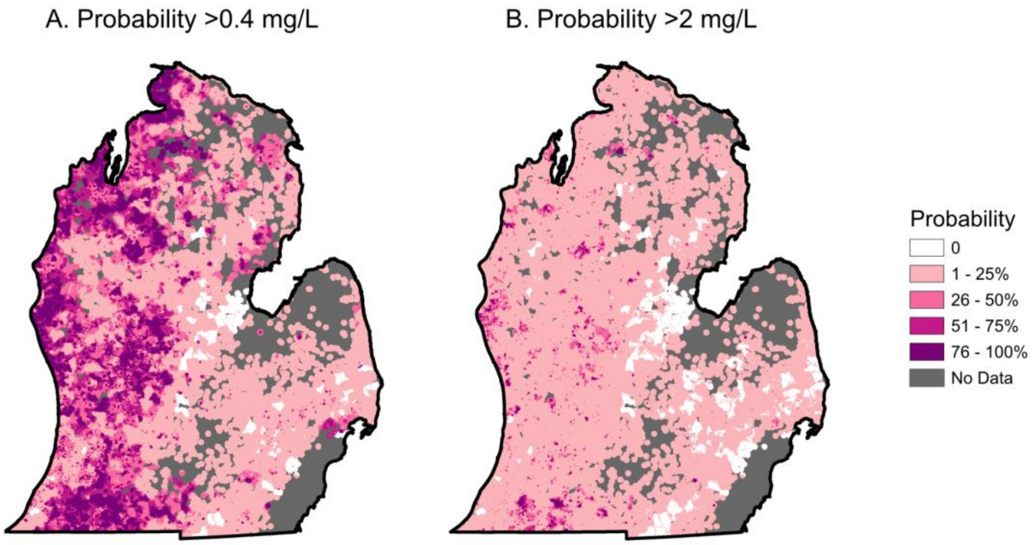

Fig S2. Continuous Probability of Exceedance Maps. These maps show intermediate steps between kriging prediction (Fig 3A) and median probability of exceedance at watershed level (Fig 3B & 3C).

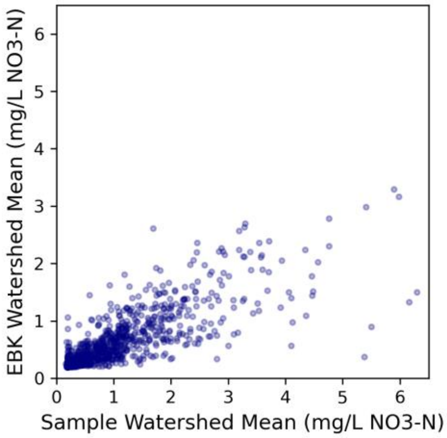

Fig S3. Comparison of observed watershed sample mean concentration and EBK-predicted watershed mean concentration.

Table S2 caption. Complete list of CART variables (see Supplemental File). Variables as described in model including description, summarizing method, and unit organized by variable category.
